# Discovery of Bioactive N-Substituted-3-methylbut-2-enamides: Synthesis, Crystal Structures, Antifungal Activity, Mechanism of Action, and Zebrafish Toxicity Evaluation

**DOI:** 10.3390/biology15212003

**Published:** 2026-11-04

**Authors:** Xuebin Zhang, Zhixuan Han, Wenjie Hu, Jiaonan Li, Jingyi Yu, Xinyu Hu, Xinjun Liao

**Affiliations:** 1 School of Life Sciences, Jinggangshan University, Ji’an 343009, China; ashangan678@163.com (X.Z.); 2408301006@jgsu.edu.cn (Z.H.); 19116208369@163.com (J.L.); 2308301006@jgsu.edu.cn (J.Y.); 2408141048@jgsu.edu.cn (X.H.); 2 School of Life Sciences, Key Laboratory of Jiangxi Province for Functional Biology and Pollution Control in Red Soil Regions, Jinggangshan University, Ji’an 343009, China

**Keywords:** N-substituted prenylamide derivatives, synthesis, structural characterization, antifungal mechanism, zebrafish, biological toxicity

## Abstract

Plant pathogenic fungi pose a serious threat to agricultural and forestry production. Long- term reliance on chemical fungicides has led to rising pathogen resistance and ecological risks, making the discovery and development of novel, safe, and highly effective antifungal compounds of great practical significance. Three N-substituted-3-methylbut-2-enamide derivatives (compounds **I**–**III**) were synthesized and structurally characterized by single-crystal X-ray diffraction, infrared spectroscopy, and elemental analysis. The minimum inhibitory concentration (MIC) values of the three compounds were determined against six common plant pathogenic fungi, and the antifungal activity was found to be governed by the N-substituent structure. Compound **III** (3-methyl-N-(4-phenoxybenzyl) but-2-enamide) was the most active, with an MIC of 25–100 mg/mL, representing a 3-fold increase over the rigid fused-ring analogs I and II, which suggests that increased molecular flexibility favors antifungal activity. Preliminary mechanistic investigation of compound **III** revealed that it induces intracellular reactive oxygen species (ROS) accumulation, compensatory upregulation of reduced glutathione, and elevated malondialdehyde levels in fungi, thereby inhibiting fungal growth through oxidative stress-mediated damage. Zebrafish embryo toxicity assays showed that compound **III** exhibited concentration- and time-dependent developmental toxicity to zebrafish embryos. The median lethal concentration (LC_50_) was 1.032 mg/L under 72 hpf exposure conditions; sublethal exposure concentrations reduced embryo survival rate, caused developmental delay, and induced neurobehavioral abnormalities. This study revealed how structural features influence the antifungal activity of this class of enamide derivatives, preliminarily elucidated the oxidative stress-mediated antifungal effect, and completed the aquatic safety evaluation, providing an experimental basis for the further structural optimization of this lead scaffold.

## Simple Summary

Fungal diseases have brought great losses to agricultural and forestry production. Many antifungal agents are facing the problems of pathogen resistance and ecological safety. Therefore, it is of great practical significance to continuously develop new antifungal active substances. In this study, three N-substituted-3-methylbut-2-enamide derivatives were synthesized: compound **I** (3-methyl-N-(1,2,3,4-tetrahydronaphthalen-1-yl)but-2-enamide), compound **II** (N-(2,3-dihydro-1H-inden-1-yl)-3-methylbut-2-enamide), and compound **III** (3-methyl-N-(4-phenoxybenzyl)but-2-enamide). Their chemical structures were unambiguously characterized. Antifungal bioassays against six plant pathogenic fungi indicated that variation in the N-substituent affected fungicidal activity; among the three compounds, compound **III** exhibited superior inhibitory activity. Further mechanistic studies demonstrated that compound **III** exerted antifungal effects by inducing fungal oxidative damage and disrupting cell membrane integrity. However, zebrafish toxicity evaluation indicated that compound **III** possessed some aquatic biotoxicity. This study provided insights into the structure–activity association and antifungal mechanism of this class of enamide compounds, offering a theoretical basis for the development of novel plant-protection fungicides. Nevertheless, further structural optimization is still required to balance antifungal efficacy and biosafety for future practical field application.

## 1. Introduction

The sustainable development of agriculture and forestry is closely related to food security and ecological security. As a major biotic stress factor threatening such development, plant fungal diseases often lead to severe yield losses and ecological damage [1,2,3]. Currently, chemical synthetic fungicides remain the primary approach to field disease control. However, their prolonged use has led to a series of issues, including increased pathogen resistance, accumulation of environmental residues, and toxicity to non-target organisms [4,5,6,7,8]. Therefore, the development of novel antifungal agents with unique structures, distinct mechanisms of action, and environmental friendliness has become an important research direction for the green and sustainable management of agricultural and forestry diseases [9,10,11].

Enamide compounds belong to a class of important organic molecules, and the skeleton contains *α*,*β*-unsaturated amide pharmacophores. In this *α*,*β*-unsaturated amide fragment, the carbon–carbon double bond acts as a Michael acceptor and can form potential covalent interactions with nucleophilic amino acid residues in proteins; the amide bond is capable of dual hydrogen-bonding interactions and can mediate molecular recognition in biological systems [12]. This kind of electrophilic double bond is one of the key structural features that determine the biological activity of *α*,*β*-unsaturated amide derivatives. Based on the above structural characteristics, this kind of molecule is easy to carry out structural modification, and the biological activity also has adjustable space. Compared with traditional antifungal drugs such as azoles and polyenes, the lipid–water partition coefficient of enamide molecules can be regulated by N-substitution or side chain modification. Previous studies have reported that after replacing the less stable coumarin ring with an *α*,*β*-unsaturated amide scaffold, the molecule exhibits a 4-fold enhancement in metabolic stability while maintaining antifungal activity, and it also shows activity against drug-resistant strains [13].

3-Methylbut-2-enamide is a representative derivative of *α*,*β*-unsaturated amide. From the perspective of organic structural theory, the *β*-methyl group can reduce the electrophilicity of the *β*-carbon via electron-donating inductive effects and hyperconjugation. Meanwhile, it alters the planarity of the conjugated system and molecular conformation through steric hindrance. Accordingly, changes in the electron distribution and reactivity of the *α*,*β*-unsaturated carbonyl system can be anticipated. Although enamides and their N-substituted derivatives have been studied extensively, with bioactivities covering antifungal [14,15], anti-inflammatory [16,17], antitumor [18,19], and insecticidal [20,21] fields, research on the synthesis and activity evaluation of the N-substituted 3-methylbut-2-enamide scaffold against plant pathogenic fungi remains very scarce. Few activity reports are available for N-substituted derivatives bearing diverse aromatic scaffolds such as tetralin, indane, and diphenyl ether, and the effects of these substituent frameworks on antifungal activity remain unclear. Currently, research on the 3-methylbut-2-enamide scaffold against plant pathogenic fungi remains insufficient, and the structure–activity relationship (SAR) by which N-position substituents regulate antifungal activity has not yet been elucidated, which to some extent limits the further development of this scaffold as an antifungal lead. Therefore, introducing different aromatic substituents at the N-position of this scaffold is expected to lead to the discovery of novel antifungal lead compounds.

Zebrafish is a widely used model aquatic organism. Its gene has high homology with human and aquatic organisms, short embryonic development cycle, and sensitive toxicity response. It is an ideal model for evaluating the ecological toxicity and environmental safety of pesticide small molecule compounds [22]. However, systematic studies on the synthesis, structure–activity relationships, antifungal mechanisms of action, and biotoxicity of N-substituted 3-methylbut-2-enamide compounds remain relatively scarce. In the preliminary stage of this project, we synthesized more than 30 types of enamide derivatives with diverse N-substituents. The substituent types included halobenzyl, 1-tetrahydronaphthyl, 1-indanyl, and 4-phenoxybenzyl, along with other moieties, and we conducted batch preliminary screening of the antifungal activities of these compounds. Based on the screening results, three molecules with favorable antifungal activity and representative N-substituent types were selected for in-depth investigation in this study. The compounds were characterized by elemental analysis, infrared spectroscopy, and nuclear magnetic resonance spectroscopy, and their molecular structures were further confirmed by X-ray single-crystal diffraction. The inhibitory effects of the above compounds on plant pathogenic fungi were determined by in vitro antifungal test. The differences in substituents of the three representative molecules were compared to observe the effect of substituent changes on antifungal activity. On this basis, the derivative with the optimal activity was screened out. Physiological and biochemical index detection and microscopic morphological observation were performed to preliminarily explore the antifungal mechanism of this compound. Meanwhile, zebrafish embryos were used as a model organism to evaluate the aquatic toxicity of the highly active compound. The results can provide basic experimental data for the early safety evaluation of this class of compounds and also serve as a reference for assessing potential environmental risks in the subsequent development of agricultural antifungal agents based on this scaffold.

## 2. Materials and Methods

### 2.1. Main Instruments and Reagents

XFS-300CB Autoclave (Shanghai Shangyi Instrument Equipment Co., Ltd., Shanghai, China); SPH-250 Biochemical Incubator (Shanghai Senxin Experimental Instrument Co., Ltd., Shanghai, China); SW-CJ Series Clean Bench (Shanghai Xinmiao Medical Device Manufacturing Co., Ltd., Shanghai, China); INVENIO Infrared Spectrometer, KBr pellet method (Bruker Optics GmbH, Ettlingen, Germany); UNICUBE Elemental Analyzer (Elementar Analysensysteme GmbH, Langenselbold, Germany); PROII X-Ray Single Crystal Diffractometer (Rigaku Corporation, Tokyo, Japan); MP50 Melting Point Apparatus (Mettler-Toledo GmbH, Greifensee, Switzerland); Avance III NMR Spectrometer (Bruker BioSpin GmbH, Rheinstetten, Germany); Ci-s upright fluorescence microscope (Nikon Corporation, Tokyo, Japan); M1000 Pro microplate reader (Tecan Group Ltd., Männedorf, Switzerland); JY92-IIN-EC ultrasonic cell disruptor (Ningbo Scientz Biotechnology Co., Ltd., Ningbo, China); ZEISS Discovery.V20 stereomicroscope (Carl Zeiss AG, Oberkochen, Germany); Eppendorf 5804R centrifuge (Eppendorf AG, Hamburg, Germany).

Wild type (AB) and Tg (elavl3: enhanced green fluorescent protein (EGFP)) transgenic zebrafish were obtained from the Chinese Zebrafish Resource Center (Wuhan, China). Zebrafish were cultured according to the breeding conditions described in the literature [23]. The animals were kept at 28 ± 0.5 °C under a 14-h light/10-h dark cycle. 1-phenyl-2-thiourea was purchased from Sigma-Aldrich (Darmstadt, Germany), and dimethyl sulfoxide was purchased from Sangon Biotech (Shanghai, China). 1,2,3,4-Tetrahydro-1-naphthylamine (purity 98%), triethylamine (purity 99%), 3-methylcrotonoyl chloride (purity 98%), 1-aminoindane (purity 98%), 4-dimethylaminopyridine (DMAP, purity 99%), 3-methylbut-2-enoic acid, 1-(3-dimethylaminopropyl)-3-ethylcarbodiimide hydrochloride (EDCI), 4-phenoxybenzylamine, methanol, dichloromethane, KBr, anhydrous ethanol, and carbendazim solution were commercially available and of analytical grade. The tested strains, including *Fusarium solani*, *Colletotrichum musae*, *Fusarium equiseti*, *Colletotrichum lagenarium*, *Fusarium heterosporum*, and *Monilinia fructigena*, were all purchased from Beijing Kezhan Biotechnology Co., Ltd. (Beijing, China). Potato dextrose broth (PDB) medium (Hopebiol, HB0233-4, Qingdao Hope Bio-Technology Co., Ltd., Qingdao, China), reactive oxygen species (ROS) assay kit (Beyotime, S0034S, Beyotime Biotechnology, Shanghai, China), hydrogen peroxide (G82427M, Sinopharm Chemical Reagent Co., Ltd., Shanghai, China), lipid peroxidation (malondialdehyde, MDA) assay kit (Beyotime, S0131S, Beyotime Biotechnology, Shanghai, China), bicinchoninic acid (BCA) protein concentration assay kit (Beyotime, P0009, Beyotime Biotechnology, Shanghai, China), reduced glutathione (GSH) assay kit (YX-C-A200, Shanghai Youxuan Biotechnology, Shanghai, China), propidium iodide (PI) staining solution (Beyotime, C1008S, Beyotime Biotechnology, Shanghai, China).

### 2.2. Experimental Methods

#### 2.2.1. Synthesis of Compounds

##### Compound **I** (3-Methyl-N-(1,2,3,4-tetrahydronaphthalen-1-yl)but-2-enamide)

**Scheme 1:**
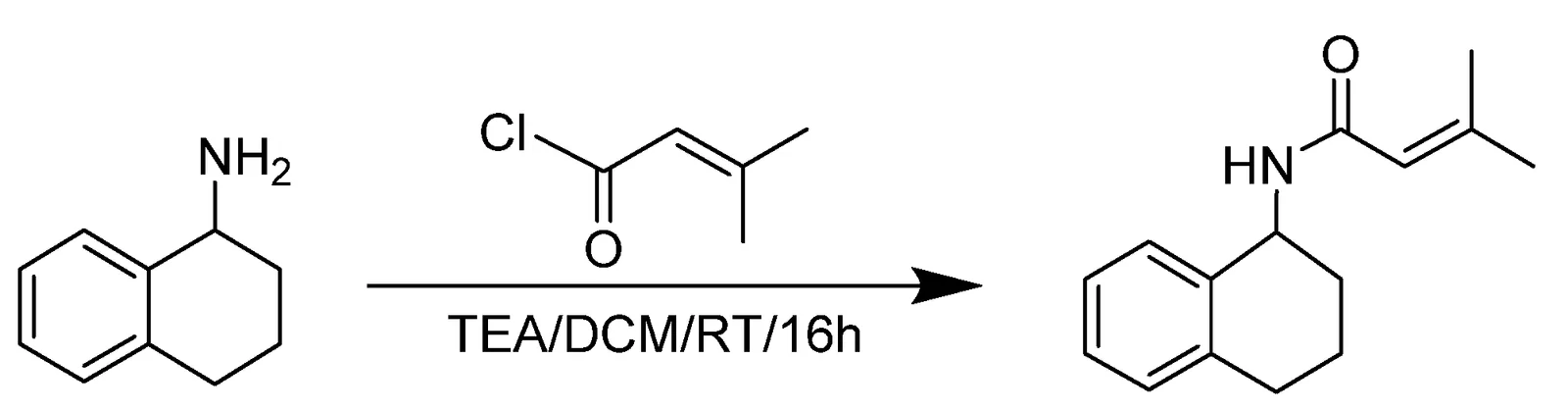
Synthesis of compound **I**.

The general procedure for the synthesis of compound **I** is shown in Scheme 1. A solution of 1,2,3,4-tetrahydro-1-naphthylamine (480 mg, 2.76 mmol) and triethylamine (1.2 mL, 8.27 mmol) in dichloromethane (20 mL) was prepared. To this solution, 3-methylcrotonyl chloride (359.77 mg, 3.03 mmol) was added dropwise at room temperature. The resulting mixture was stirred at the same temperature for 16 h. After the reaction was complete, the mixture was successively washed with water (20 mL) and brine. The organic layer was dried over anhydrous sodium sulfate, filtered, and concentrated under reduced pressure to give the crude product, which was purified by column chromatography (eluent: petroleum ether/ethyl acetate, 93:7) to afford the title compound as a white solid (160 mg, yield 25.3%).

Analytical data for compound **I** White solid; m.p.: 115.7–117.1 °C. ^1^H NMR (400 MHz, CDCl_3_) *δ* 7.34–7.26 (m, 1H), 7.21–7. 12 (m, 2H), 7.11–7.08 (m, 1H), 5.65–5.49 (m, 2H), 5.28–5.16 (m, 1H), 2.86–2.70 (m, 2H), 2.20 (d, *J* = 0.9 Hz, 3H), 2.11–2.00 (m, 1H), 1.88–1.77 (m, 6H). ^13^C NMR (126 MHz, CDCl_3_) *δ* 166.19, 151.11, 137.63, 136.99, 129.14, 128.86, 127.20, 126.24, 118.61, 77.32, 77.06, 76.81, 46.97, 30.27, 29.29, 27.21, 20.00, 19.85. ESI-MS *m*/*z*: 230.2 [M + H]^+^. All NMR spectra are provided in Supplementary File S1.

##### Compound **II** (N-(2,3-Dihydro-1H-inden-1-yl)-3-methylbut-2-enamide)

**Scheme 2:**
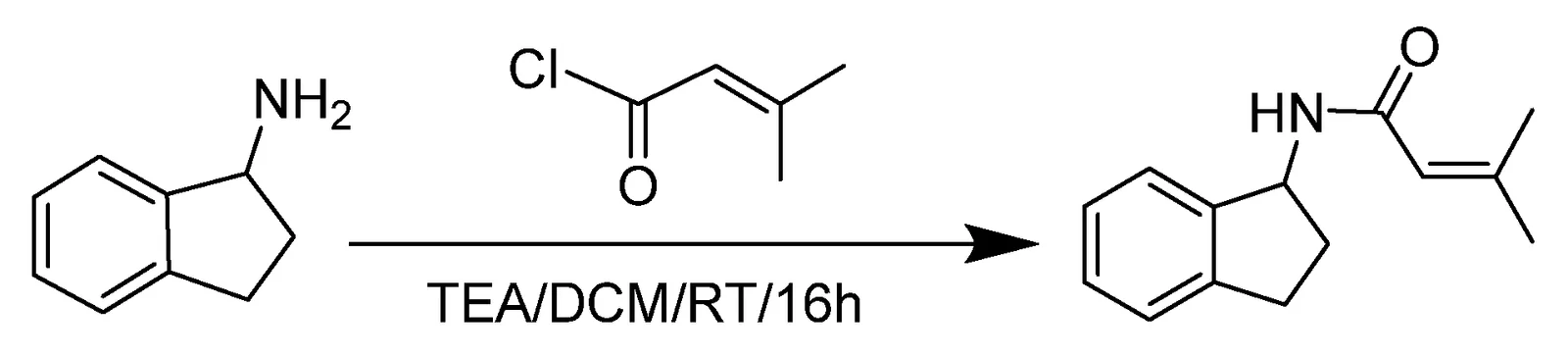
Synthesis of compound **II**.

The general procedure for the synthesis of compound **II** is shown in Scheme 2. A mixture of 1-aminoindane (500 mg, 3.76 mmol) and triethylamine (1.5 mL, 11.27 mmol) in dichloromethane (20 mL) was treated dropwise with 3-methylcrotonyl chloride (490.12 mg, 4.14 mmol) at room temperature. The resulting mixture was stirred at the same temperature for 16 h. After completion of the reaction, the reaction mixture was successively washed with water (20 mL) and brine. The organic layer was dried over anhydrous sodium sulfate, filtered, and concentrated under reduced pressure. The crude product was purified by column chromatography (eluent: petroleum ether/ethyl acetate, 93:7) to afford the title compound as a white solid (350 mg, yield 43.3%).

Analytical data for compound **II** White solid; m.p.: 105.1–107.7 °C. ^1^H NMR (400 MHz, CDCl_3_) *δ* 7.37–7.13 (m, 4H), 5.61 (d, *J* = 7.2 Hz, 1H), 5.56 (dt, *J* = 2.5, 1.2 Hz, 1H), 5.52 (dd, *J* = 15.4, 7.7 Hz, 1H), 3.02–2.94 (m, 1H), 2.91–2.82 (m, 1H), 2.66–2.53 (m, 1H), 2.20 (d, *J* = 1.2 Hz, 3H), 1.86–1.77 (m, 4H). ^13^C NMR (126 MHz, CDCl_3_) *δ* 166.72, 151.26, 143.48, 127.89, 126.74, 124.79, 124.08, 118.46, 77.33, 77.07, 76.82, 54.28, 34.26, 30.28, 27.22, 19.86. ESI-MS *m*/*z*: 216.2 [M + H]^+^. All NMR spectra are provided in Supplementary File S1.

##### Compound **III** (3-Methyl-N-(4-phenoxybenzyl)but-2-enamide)

Compounds **I** and **II** were synthesized by the acyl chloride route (method A), and compound **III** was prepared by mixed anhydride/CDI condensation (method B). Both routes are mature methods for constructing amide bonds. For compound **III**, method B was selected because of its simpler post-processing and purification operations. The highly active intermediate generated by method A is easy to cause side reactions such as excessive acylation and hydrolysis. The intermediate activity of the mixed anhydride/CDI system is moderate, which is conducive to the selective nucleophilic attack of amines. This study focused on the evaluation of antifungal activity. Method B was used to quickly obtain sufficient samples for biological screening, and no systematic comparison and condition optimization were performed on the two routes.

**Scheme 3:**
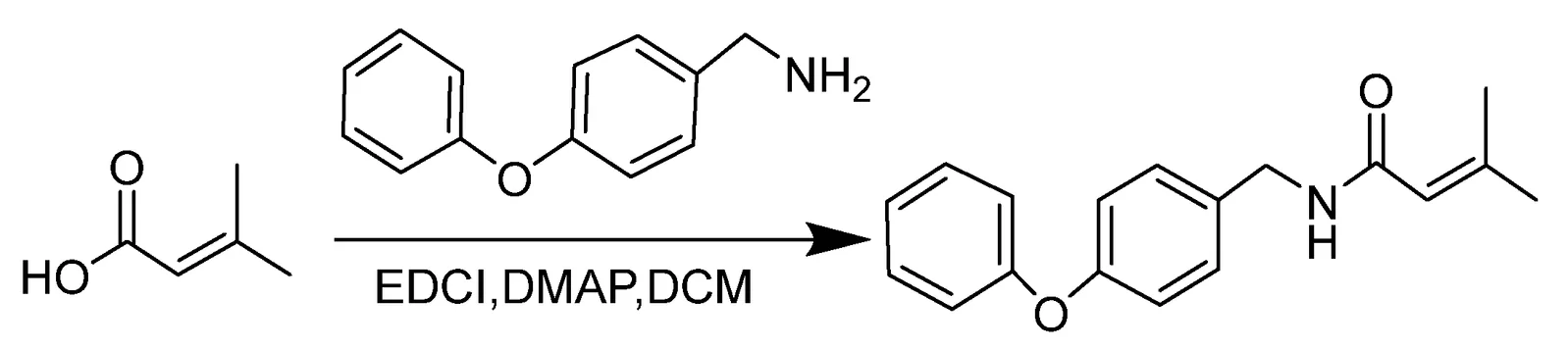
Synthesis of compound **III**.

The general procedure for the synthesis of compound **III** is shown in Scheme 3. A mixture of 3-methylbut-2-enoic acid (400 mg, 4.0 mmol), EDCI (920 mg, 4.8 mmol), and DMAP (244 mg, 2.0 mmol) in dichloromethane (20 mL) was stirred for 15 min. To this mixture, 4-phenoxybenzylamine (956 mg, 4.8 mmol) was added. The reaction was then stirred at room temperature for 16 h. Upon completion, the mixture was successively washed with water (20 mL) and brine. The organic layer was dried over anhydrous sodium sulfate, filtered, and concentrated under reduced pressure to afford the crude product, which was purified by column chromatography (eluent: petroleum ether/ethyl acetate, 90:10) to give the title compound as a white solid (480 mg, yield 42.6%).

Analytical data for compound **III** White solid; m.p.: 83.2–96.9 °C. ^1^H NMR (400 MHz, CDCl_3_) *δ* 7.35–7.28 (m, 2H), 7.27–7.21 (m, 2H), 7.12–7.06 (m, 1H), 7.01–6.92 (m, 4H), 5.79 (b, 1H), 5.62–5.52 (m, 1H), 4.42 (d, *J* = 5.8 Hz, 2H), 2.18 (d, *J* = 1. 1 Hz, 3H), 1.83 (d, *J* = 1. 1 Hz, 3H). ^13^C NMR (126 MHz, CDCl_3_) *δ* 166.84, 157.20, 156.49, 151.60, 133.58, 129.78, 129.28, 123.30, 119.06, 118.80, 118.25, 42.65, 27.25, 19.88. ESI-MS *m/z*: 282.2 [M + H]^+^. All NMR spectra are provided in Supplementary File S1.

#### 2.2.2. Single Crystal X-Ray Diffraction Analysis of the Compounds

Single crystals of compounds **I**, **II**, and **III** were grown via slow evaporation at ambient temperature from a 1:1 (*v*/*v*) mixture of methanol and dichloromethane. X-ray diffraction data were collected at 150 K (I, III) and 120 K (II) on a Rigaku Pro II diffractometer with Cu Kα radiation (*λ* = l.54l84 Å) in ω-scan mode. Data integration and reduction were performed using the CrysAlisPro software (version 43.143a), and semi-empirical absorption corrections were carried out via the multi-scan method implemented in the same software. The structures were solved by direct methods using Olex2 software (version 1.2) with the olex2.solve program and refined by full-matrix least-squares on *F*^2^ with the ShelXL (version 2018/3) software package. All non-hydrogen atoms were refined anisotropically. Hydrogen atoms were placed at calculated positions and refined isotropically using a riding model.

#### 2.2.3. Infrared Spectroscopy Analysis

The thermally stable sample was vacuum-dried at 40–60 °C for several hours. Meanwhile, KBr powder was dried in an oven at 110 °C for at least 2 h, cooled, and stored in a desiccator for subsequent use. The sample and KBr were weighed precisely in a mass ratio of 1:100 to 1:200. Under dry conditions, the sample was first ground to a particle size below 2 μm. KBr was then added, and the mixture was co-ground for 5–10 min until it became homogeneous and translucent. The mixed powder was then promptly transferred to a clean, dry pellet die. After gently tapping the die to distribute the powder evenly, the pellet was pressed, ejected with a dedicated rod, and picked up with tweezers. The prepared pellet was placed in the FTIR sample holder. A background scan was performed first, followed by the sample measurement. The spectral data were collected over a range of 4000–400 cm^−1^ with a resolution of 4 cm^−1^ and 32 accumulated scans.

#### 2.2.4. Elemental Analysis Determination

Elemental analysis (C, H, N, O) was performed using the Elementar UNICUBE elemental analyzer. The sample was ground into uniform fine powder and fully dehydrated by vacuum drying or freeze drying. Based on the sensitivity of the instrument and the estimated content of the elements to be measured, 1–5 mg of dry samples was weighed using an ultramicrobalance with an accuracy of 0.001 mg. For the determination of C, H, and N, the sample was placed in a tin capsule, folded, and sealed, and its exact mass was recorded. Benzoic acid, acetanilide, and other reference materials were used for multi-point instrument calibration. Blank tin capsules were analyzed simultaneously to eliminate background interference. The standard sample was determined in parallel for 3 times to ensure that the relative standard deviation (RSD) of the results was less than 0.3%. The sealed sample capsules were loaded into the autosampler and automatically introduced into the high-temperature furnace for combustion analysis. The oxygen content was separately determined on the same instrument by switching to the oxygen pyrolysis mode: the sample was pyrolyzed at high temperature, and organic oxygen was converted into carbon monoxide (CO), which was quantified using a TCD detector. The determined elements and their mass percentages are as follows: compound **I** (C: calculated 78.60%, found 78.45%; N: calculated 6.11%, found 6.16%; H: calculated 8.30%, found 7.48%; O: calculated 6.99%, found 7.23%); compound **II** (C: calculated 78.08%, found 78.08%; N: calculated 6.51%, found 6.55%; H: calculated 7.91%, found 7.92%; O: calculated 7.44%, found 7.46%); compound **III** (C: calculated 76.87%, found 76.54%; N: calculated 4.98%, found 5.02%; H: calculated 6.76%, found 6.66%; O: calculated 11.39%, found 11.62%).

#### 2.2.5. Determination of Antifungal Activity

##### Cultivation of Strain

*F. solani*, *C. musae*, *F. equiseti*, *C. lagenarium*, *F. heterosporum*, and *M. fructigena* were used as the tested strains. They were inoculated onto potato dextrose agar (PDA) solid medium plates and incubated in a constant temperature incubator at 28 °C for 3–5 d. The plates were ready for use after the mycelia fully covered the whole plates.

##### Determination of the MIC

The antifungal activities of the three compounds against different fungi were evaluated using the mycelial growth inhibition method [24,25,26] and the filter paper strip diffusion method [27]. Using absolute ethanol as the solvent, stock solutions of each compound were prepared at mass concentrations of 100, 50, 25, 12.5, and 6.25 mg/mL. Sterile filter paper strips (6 cm × 6 mm) were separately immersed in compound solutions of different concentrations until the strips were fully saturated. In a laminar flow cabinet, sterilized PDA medium was poured into Petri dishes. The filter paper strips impregnated with the compound solution were removed, air-dried appropriately, and then affixed onto the surface of the same agar plate in ascending order of concentration. Filter paper strips were placed in parallel with a spacing of 1.5 cm to prevent potential overlap of diffusion zones. Mycelial plugs (5 mm in diameter) of the test fungi were inoculated at the center of each plate, with three replicate plates per treatment.

All plates were incubated at 28 °C for 10 days. Fungal growth was continuously monitored visually during incubation. The antifungal results were recorded before the drug diffusion zones fully merged, to accurately identify the concentration boundary that halted the advancing mycelial front. Accordingly, the MIC was operationally defined as the lowest concentration of solution loaded on filter paper strips that completely blocked further expansion of the mycelial front. After incubation, fungal growth around each filter paper strip was observed and recorded. The antifungal efficacy of the compounds was assessed based on the degree of mycelial growth inhibition surrounding the strips. Negative controls (absolute ethanol) and positive controls (carbendazim, evaluated at the identical concentration gradients of 100, 50, 25, 12.5, and 6.25 mg/mL) were included to account for any solvent effects and validate the experimental system.

#### 2.2.6. Determination of Physiological and Biochemical Indices Related to Oxidative Stress in *F. heterosporum* Treated with Compound **III**

##### Detection of ROS

Five mL of *F. heterosporum* culture in the logarithmic growth phase was collected and centrifuged at 4000 rpm for 10 min in an Eppendorf 5804R centrifuge. The supernatant medium was discarded, and the pellet was resuspended in 1 mL of fresh PDB medium (Haibo Biotechnology, Ningbo, China, Cat. No. HB0233-4). An aliquot of 1 mL was taken from each overnight stored PDB-based drug solution (final concentrations: 2.5 mg/mL and 1.25 mg/mL) and 4 mM H_2_O_2_ (Sinopharm Chemical Reagent Co., Ltd., Cat. No. G82427M). Subsequently, 200 μL of fungal suspension was added to each solution. After mixing by gentle inversion, the mixtures were incubated at 30 °C for 2 h, 4 h, and 8 h, with three biological replicates for each concentration and each time point. The dynamic changes of ROS were evaluated in time-gradient assays. Combined with preliminary experimental results, the treatment duration for subsequent mechanistic assays was set to 4 h. Two controls were included: a solvent control with 1% dimethyl sulfoxide (DMSO) containing fungal cells, and a drug containing medium blank control without fungal cells. After incubation, the samples were centrifuged and washed once with phosphate-buffered saline (PBS), with the pellet resuspended in 1 mL of fresh PBS. Intracellular ROS were measured according to the protocol for suspended cells provided by the ROS Assay Kit (Beyotime, Cat. No. S0034S).

##### Determination of Lipid Peroxidation Damage (MDA)

Fungal cell preparation, drug solutions, and control groups were performed as described above for the intracellular ROS assay. In this assay, logarithmic-phase *F. heterosporum* cells were exposed to compound **III** (1.25 mg/mL and 2.5 mg/mL) and 4 mM H_2_O_2_ at 30 °C for 4 h only, with three biological replicates for each concentration. Following incubation, the samples were centrifuged and washed once with PBS. The supernatant was discarded to harvest fungal pellets. The lysis buffer provided by the kit was added to the fungal pellets, followed by physical disruption using an ultrasonic homogenizer. After sonication, the mixture was centrifuged at 12,000 rpm for 10 min to collect the clear lysate supernatant, and an aliquot of the lysate supernatant was used to determine MDA content according to the protocol of the MDA assay kit (Beyotime, Cat. No. S0131S). At the same time, the supernatant of the other part of the lysate was used to detect the protein content with the bicinchoninic acid (BCA) protein concentration determination kit (Biyuntian, P0009) for the calculation of MDA.

##### Detection of Reduced Glutathione (GSH)

An aliquot of the same lysate used for MDA detection was employed to measure GSH content following the protocol of the GSH assay kit (Shanghai Youxuan Biotechnology, Cat. No. YX-C-A200). GSH levels were normalized against the corresponding total protein content.

##### Cell Membrane Integrity (PI Staining) Detection

The fungus suspension was prepared according to the above method. After incubation, the samples were centrifuged and washed once with PBS, and they were then resuspended in 1 mL of fresh PBS. PI staining solution (Beyotime, C1008S) was added to a final concentration of 5 μg/mL. Following 15 min incubation at room temperature, the mixture was homogenized, and 200 μL of the fungus suspension was transferred onto a glass slide and covered with a coverslip. Images were acquired under a 200-fold magnification using an upright fluorescence microscope (Nikon, Ci-s) in the red fluorescence channel and bright-field mode.

#### 2.2.7. Zebrafish Embryo Toxicity Test and Developmental Phenotype Observation

Compound **III** was dissolved in DMSO to prepare a stock solution (5 mg/mL) and stored at −20 °C. One healthy and sexually mature female and one male were selected from the aquarium and placed in a mating tank. The next morning, the fertilized eggs were transferred to a culture dish containing embryonic medium (5 mM NaCl, 0.17 mM KCl, 0.33 mM CaCl_2_, 0.33 mM MgSO_4_) [22]. At 6 h post-fertilization (hpf), normally developed zebrafish embryos were randomly selected and distributed into a 6-well plate (20 embryos per well). Each well was filled with 5 mL of embryo medium containing 0.003% 1-phenyl-2-thiourea (PTU) to inhibit pigmentation. The concentration gradients were 0, 0.1, 0.3, 0.5, 0.7, 0.9, and 1.1 mg/L, respectively, and the exposure was carried out according to the organisation for economic co-operation and development (OECD) fish embryo acute toxicity test (FET) guidelines [22]. The overall survival rate was used to determine the optimal exposure concentration. Finally, the exposure concentration of compound **III** was selected as 0.3, 0.5, and 0.7 mg/L, respectively. The control group was exposed to 0.05% DMSO solvent, while all compound **III** treatment groups maintained the same DMSO concentration. All exposure solutions were renewed every 24 h.

#### 2.2.8. Morphological Observation

After exposure to compound **III**, the mortality and hatching rate of zebrafish embryos were observed under a stereomicroscope at 24, 48 and 72 h post fertilization (hpf), respectively. The criteria for embryonic death were as follows: egg coagulation, somite loss, yolk sac detachment, or heartbeat disappearance. The number of hatched and unhatched larvae was counted at 72 hpf. At 72 hpf, a ZEISS stereomicroscope (Discovery.V20, Oberkochen, Germany) was used to observe and photograph the morphological characteristics of juvenile fish. The main toxic phenotypes included pericardial edema and tail deformation. Subsequently, the larvae were anesthetized with 0.168 g/L tricaine (MS-222), and The bright-field and fluorescence images were captured using a ZEISS Discovery.V20 stereo fluorescence microscope. The Tg (elavl3: EGFP) transgenic fish line was used to label neurons and perform fluorescence quantification [28]; at the same time, the body length and pericardial area of juvenile fish were measured using ZEISS ZEN (version 3.8).

#### 2.2.9. Determination of Zebrafish Behavior

Compound **III** exposure was performed from 6 to 72 hpf, and the whole experiment was conducted at room temperature. Thereafter, larvae were maintained in fresh embryo medium (E3) from 72 to 144 hpf with daily medium changes. On day 6 (144 hpf), larvae were individually placed into the wells of a 24-well plate containing 1 mL of fresh embryo medium per well, and the plate was placed in a DanioVision tracking system (Noldus, The Netherlands) and left undisturbed for 20 min before locomotor activity recording. The recording protocol consisted of alternating light and dark phases (2 min light/2 min dark, five cycles), following established methods [29,30,31]. Activity frequency, mean velocity, total distance moved, maximum acceleration, and cumulative duration of movement were directly extracted using EthoVision XT (version 15) without applying any activity threshold.

#### 2.2.10. Statistical Analysis

All experiments were performed with at least three independent biological replicates. Quantitative data are expressed as the mean ± standard deviation (SD). Statistical analyses were conducted using SPSS 27. One-way analysis of variance (ANOVA) was performed, followed by Dunnett’s post hoc test for intergroup comparisons against the control group. Asterisks denote statistically significant differences relative to the control group (* *p* < 0.05, ** *p* < 0.01, *** *p* < 0.001). GraphPad Prism 9.5 and OriginPro 2024b were used for data visualization.

## 3. Results

### 3.1. Synthesis and Characterization

Compounds **I** and **II** were synthesized by reacting 1,2,3,4-tetrahydro-1-naphthylamine or 1-aminoindane, respectively, with 3-methylcrotonyl chloride in dichloromethane, using triethylamine as a base. The reactions were conducted at room temperature for 16 h. Compound **III** was prepared under analogous conditions by coupling 3-methylbut-2-enoic acid with 4-phenoxybenzylamine, with EDCI and DMAP as coupling agents. Upon completion, each reaction mixture was sequentially washed with water and brine, dried over anhydrous sodium sulfate, and concentrated under reduced pressure. The crude products were purified by silica gel column chromatography, using 7% ethyl acetate in petroleum ether for compounds **I** and **II** and 10% ethyl acetate in petroleum ether for compound **III**, to afford the respective target compounds.

### 3.2. Analysis of the Crystallographic Structure Compounds **I**, **II**, and **III**

**Figure 1:**
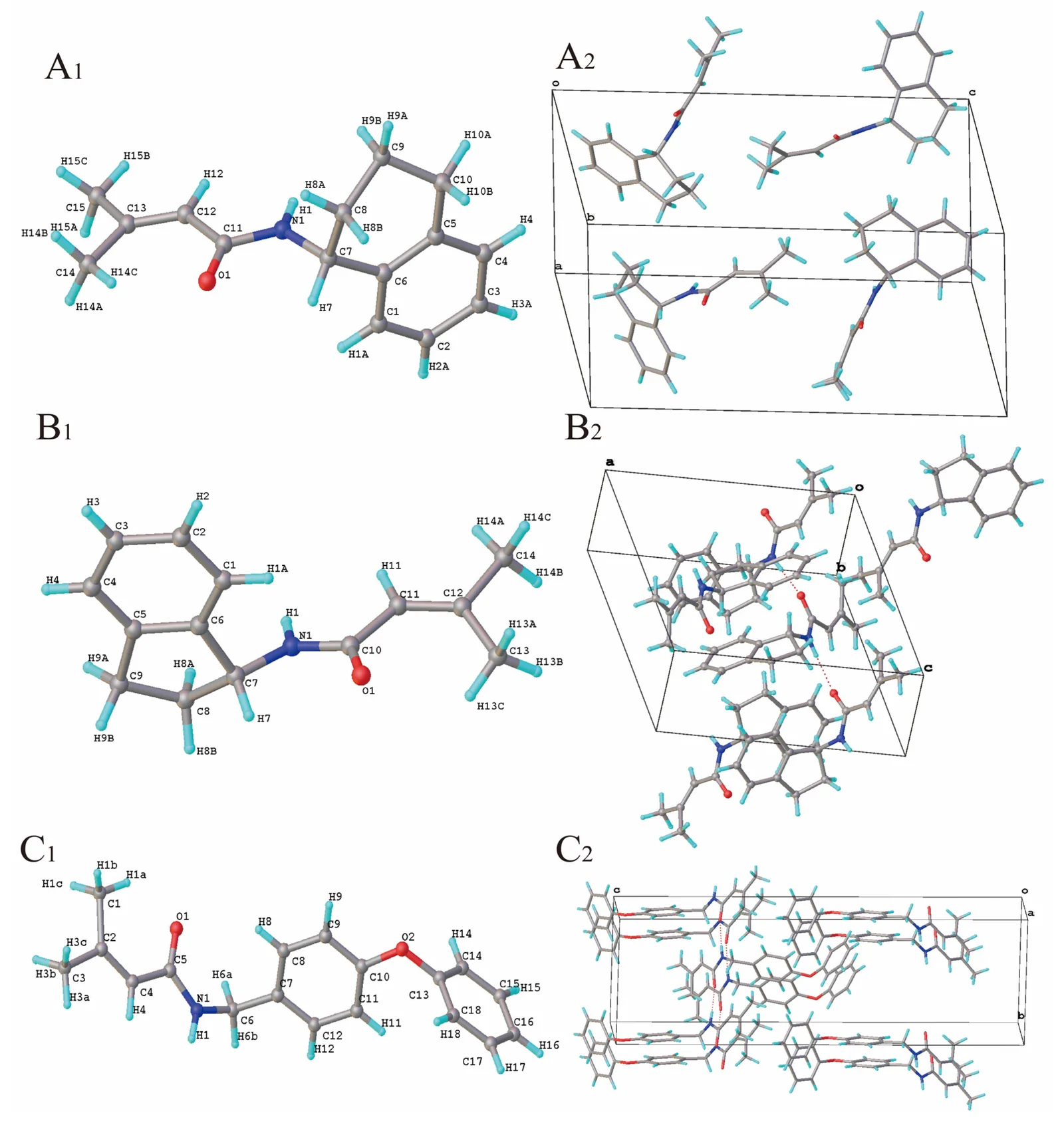
Crystal structures and crystal packing diagrams of compounds **I**, **II** and **III**. (**A_1_**), (**B_1_**) and (**C_1_**) show the molecular structures of compounds **I**, **II** and **III** with atomic numbering, respectively; (**A_2_**), (**B_2_**) and (**C_2_**) are the corresponding crystal packing diagrams. Carbon (C) atoms are grey, nitrogen (N) atoms blue, oxygen (O) atoms red, and hydrogen (H) atoms cyan. Letter O denotes the origin of the coordinate system, and a, b and c are the unit-cell axes.

Structural analysis based on single-crystal X-ray diffraction data indicates that compounds **I**, **II**, and **III** crystallize in the triclinic (space group P1), monoclinic (space group P21/c), and orthorhombic (space group P212121) systems, respectively, with unit-cell parameters as follows: compound **I**: *a* = 8.9086(4) Å, *b* = 8.9141(5) Å, *c* = 18.5488(8) Å, *α* = 79.773(4)°, *β* = 87.487(4)°, *γ* = 64.076(5)°, *Z* = 4, *D*c = 1.169 g · cm^−3^, *V* = 1302.80(12) Å^3^; compound **II**: *a* = 11.6805(3) Å, *b* = 11.3228(2) Å, *c* = 9.7294(2) Å, *α* = 90°, *β* = 114.000(3)°, *γ* = 90°, *Z* = 4, *D*c = 1.216 g ·cm^−3^, *V* = 1175.53(5) Å^3^; compound **III**: *a* = 5.47422(7) Å, *b* = 9.58669(12)Å, *c* = 29.2280(4) Å, *α* = 90°, *β* = 90°, *γ* = 90°, *Z* = 4, *D*c = 1.214 g · cm^−3^, *V* = 1533.88(3) Å^3^ (for information about crystal data and details of the structure determination, see Table 1). The refinement quality indicators (*R*int = 0.0658 for compound **I**, 0.0132 for compound **II**, and 0.0l57 for compound **III**) confirm the high reliability of the structural models. The chemical formulas derived from structural analysis are C_15_H_19_NO for compound **I**, C_14_H_17_NO for compound **II**, and C_18_H_19_NO_2_ for compound **III**, and their spatial structures are shown in Figure 1. The molecular weights calculated from these formulas are consistent with the values determined by single-crystal analysis and agree well with the mass spectrometry results: compound **I**—calculated 229.32, found 230.2; compound **II**—calculated 2l5.29, found 2l6.2; compound **III**—calculated 28l.35, found 282.2. All crystallographic data are provided in Supplementary File S2.

### 3.3. Infrared Spectral Assignment of the Compound

**Figure 2:**
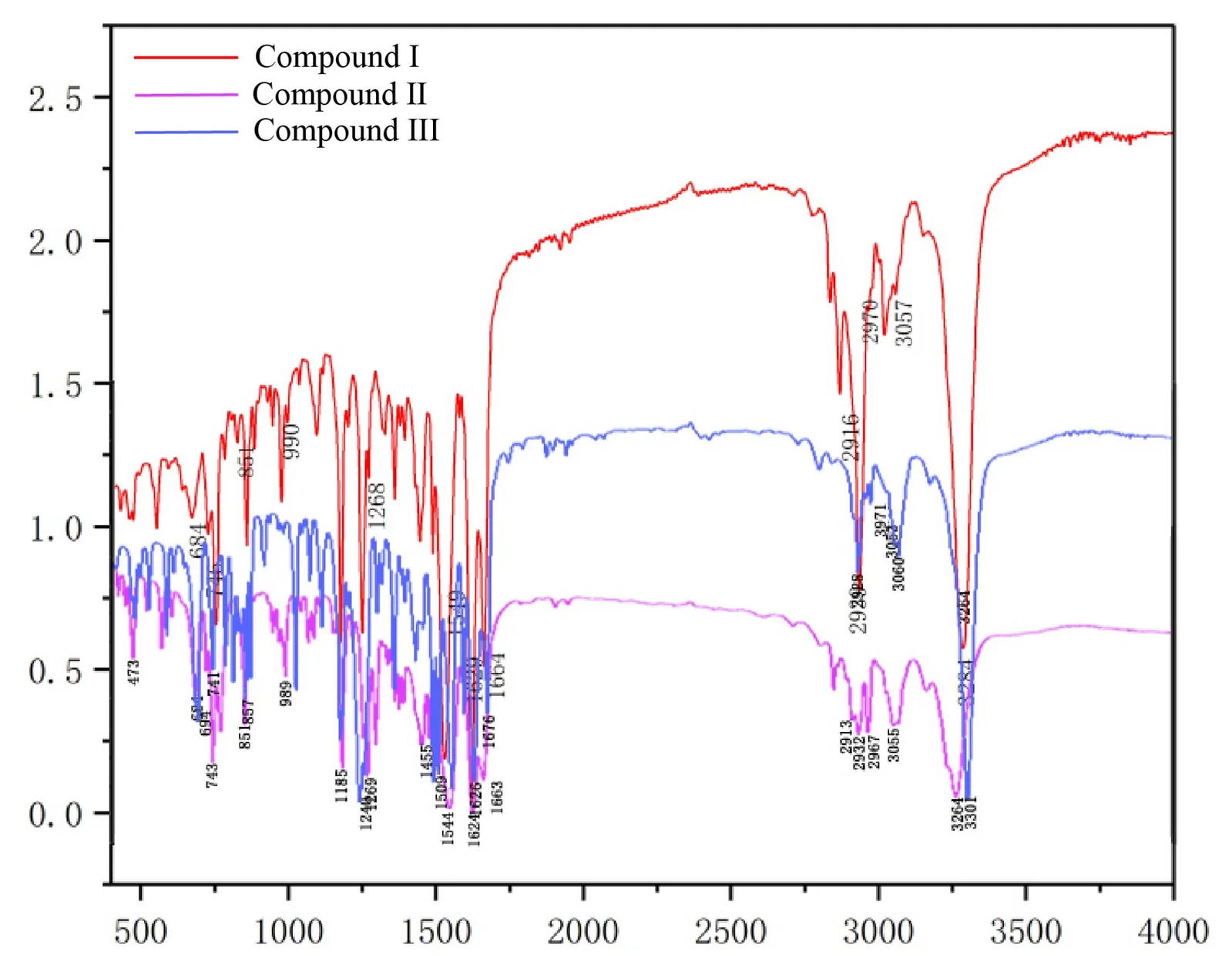
The infrared spectrum of the compounds (**I**, **II**, **III**). The numbers on the peaks indicate wavenumbers (cm^−1^) of the infrared absorption peaks.

As shown in the Figure 2, the characteristic peaks in the infrared spectrum of compound **I** are assigned as follows: the broad peaks at 3284 cm^−1^ and 1549 cm^−1^ correspond to the N–H stretching and bending vibrations, respectively; the peak at 3057 cm^−1^ is attributed to the aromatic C–H stretching vibration; the peaks at 2970 cm^−1^, 2939 cm^−1^, and 2916 cm^−1^ are assigned to the stretching vibrations of CH_2_–CH_2_–CH_2_ and =C–CH_3_; the peaks at 1664 cm^−1^ and 1268 cm^−1^ are due to C=O vibrations; the peak at 1629 cm^−1^ corresponds to the aromatic C=C stretching vibration; the peak at 990 cm^−1^ is assigned to the =C–H bending vibration; and the peaks at 851 cm^−1^, 746 cm^−1^, and 684 cm^−1^ are attributed to the bending vibrations of aromatic C=C–H.

The characteristic peaks in the infrared spectrum of compound **II** are assigned as follows: the broad peaks at 3264 cm^−1^ and 1544 cm^−1^ are attributed to the stretching and bending vibrations of N–H; the peak at 3055 cm^−1^ is attributed to the stretching vibration of aromatic =C–H; the peaks at 2967 cm^−1^, 2932 cm^−1^ and 2913 cm^−1^ are attributed to the stretching vibrations of CH_2_–CH_2_–CH_2_ and =C–CH_3_; the peaks at 1663 cm^−1^ and 1269 cm^−1^ are attributed to the vibrations of C=O; the peak at 1624 cm^−1^ is attributed to the stretching vibration of aromatic C=C; the peak at 989 cm^−1^ is attributed to the bending vibration of olefinic =C–H; and the peaks at 85l cm^−1^, 743 cm^−1^ and 684 cm^−1^ are attributed to the bending vibrations of aromatic C=C–H.

The characteristic peaks in the infrared spectrum of compound **III** are assigned as follows: the peaks at 3301 cm^−1^ and 3264 cm^−1^ correspond to N–H stretching and bending vibrations, respectively; the peaks at 3060 cm^−1^ and 3053 cm^−1^ are attributed to aromatic C–H stretching vibrations; the peaks at 2971 cm^−1^ and 2928 cm^−1^ are assigned to the stretching vibrations of saturated alkyl groups, N–CH_2_, and =C–CH_3_; the peak at 1676 cm^−1^ is due to the C=O stretching vibration; the peak at 1626 cm^−1^ corresponds to the aromatic C=C stretching vibration; the peak at 1509 cm^−1^ is attributed to N–H bending vibration; the peak at 1240 cm^−1^ corresponds to the aromatic ether C–O–C stretching vibration; and the peaks at 857 cm^−1^, 741 cm^−1^, and 694 cm^−1^ are assigned to the bending vibrations of aromatic C–H.

### 3.4. Determination of MICs of the Compounds Against Six Fungi

**Figure 3:**
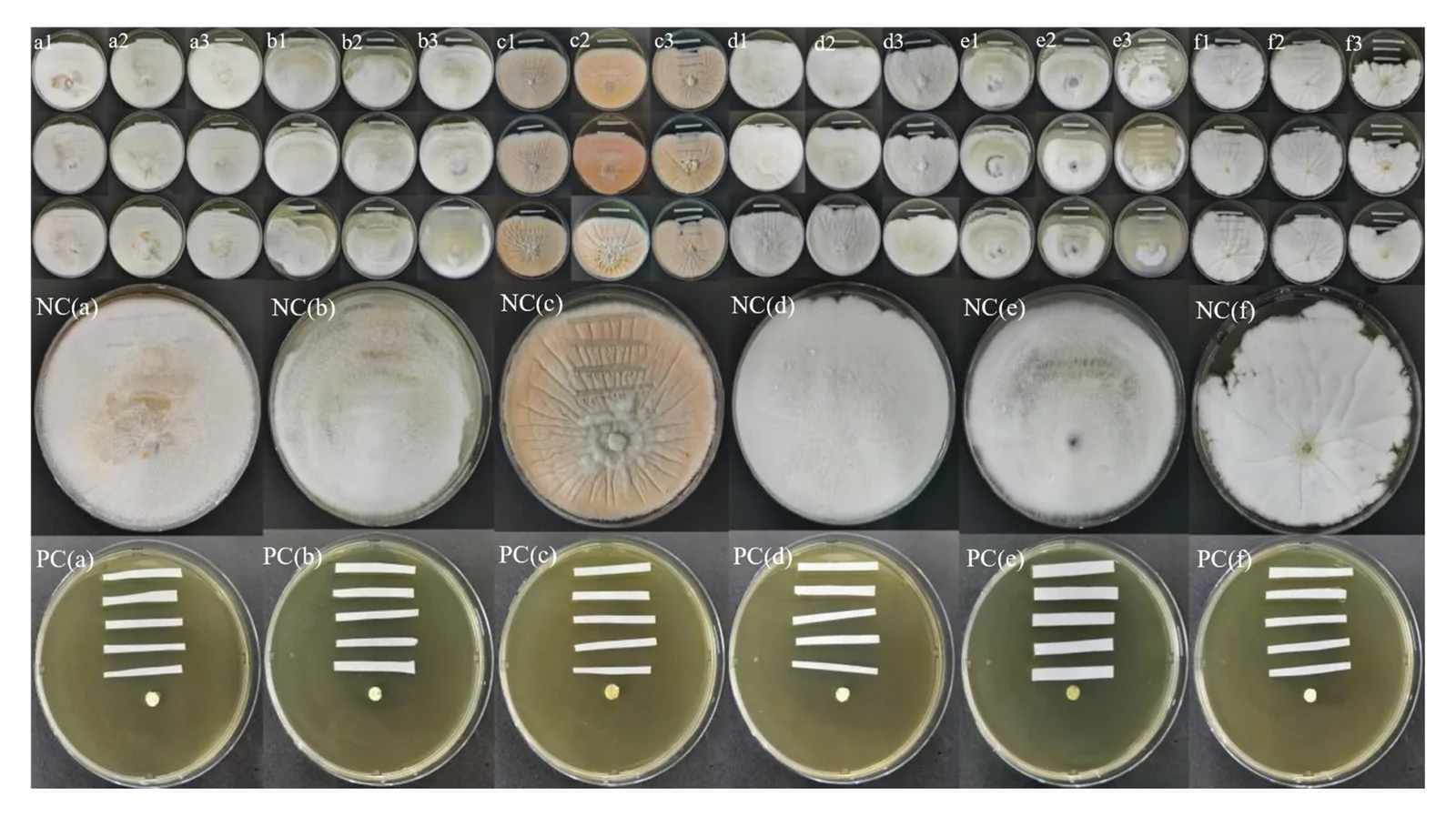
Inhibitory effects of three compounds on the mycelial growth of six fungi. Note: (**a**) *F. solani*; (**b**) *M. fructigena*; (**c**) *C. lagenarium*; (**d**) *F. equiseti*; (**e**) *C. musae*; (**f**) *F. heterosporum*. (**a1**–**f1**), (**a2**–**f2**), and (**a3**–**f3**) indicate the antifungal effects of compounds **I**, **II**, and **III** against the six pathogenic fungi, respectively. NC (**a**–**f**) and PC (**a**–**f**) represent the negative control (anhydrous ethanol on filter discs) and positive control (carbendazim on filter discs), respectively, showing the corresponding fungal colony growth on the plates.

This study assessed the antifungal activity of compounds **I**, **II**, and **III** against six plant pathogenic fungi using the mycelial growth inhibition and agar disc diffusion methods. Carbendazim was used as the positive control and evaluated with the same concentration gradient (6.25–100 mg/mL). It completely inhibited the mycelial growth of the tested fungus at all tested concentrations, indicating that its actual MIC was less than 6.25 mg/mL. Although a lower concentration gradient was not included to determine the precise MIC, the antifungal results of the positive control were consistent with expectations, verifying the reliability of this MIC assay system. The MIC results of the three compounds are shown in Table 2 and Figure 3, within the concentration range of 6.25–100 mg/mL, the MIC of compound **I** against *F. solani*, *C. musae*, *F. equiseti*, *F. heterosporum*, *M. fructigena*, and *C. lagenarium* was consistently 100 mg/mL. For compound **II**, the corresponding MICs were 100, 100, 50, 100, 100, and 100 mg/mL; for compound **III**, they were 100, 100, 50, 25, 100, and 50 mg/mL. These results demonstrate significant antifungal activity for all three compounds, with efficacy increasing in a concentration-dependent manner. Compound **III** exhibited the strongest inhibitory effect. Further integrated analysis of Table 2 and Figure 3 reveals the following against *C. lagenarium*: the antifungal efficacy of compound **III** increased with concentration from 25 to 50 mg/mL (panel c3), with the MIC established as 50 mg/mL. We used the same conditions, 100 mg/mL for compound **I** (100 mg/mL, panel c1), and 100 mg/mL for compound **II** (100 mg/mL, panel c2), respectively. For *F. equiseti*, the MICs were 50 mg/mL for compound **III** (50–100 mg/mL, panel d3), 100 mg/mL for compound **I** (100 mg/mL, panel d1), and 50 mg/mL for compound **II** (50–100 mg/mL, panel d2). Against *F. heterosporum*, the MICs were 25 mg/mL for compound **III** (25–50 mg/mL, panel f3), 100 mg/mL for compound **I** (100 mg/mL, panel f1), and 100 mg/mL for compound **II** (100 mg/mL, panel f2).

### 3.5. The Inhibitory Activity and Preliminary Structural Comparison of Three Compounds Against Tested Pathogenic Fungi

Compounds (**I**, **II**, **III**) all have the same 3-methyl-2-buteneamide parent nucleus, and the difference in antifungal activity mainly comes from the substituent groups (tetrahydronaphthalene-1-yl, dihydroinden-1-yl, and 4-phenoxybenzyl) attached to the amide nitrogen. The amide nitrogen of compound **I** is directly connected to the six-membered alicyclic fused tetrahydronaphthalene structure. The MIC of compound **I** against six tested fungi (*F. solani*, *C. musae*, *F. equiseti*, *F. heterosporum*, *M. fructigena*, *C. lagenarium*) is 100 mg/mL, which is the weakest among the three. However, compound **II** was obtained by replacing the six-membered tetrahydronaphthalene fused ring with a five-membered indane structure. The change of this structure improved the inhibitory effect of *F. equiseti* to a certain extent, and the MIC decreased from 100 mg/mL to 50 mg/mL. It can be seen that in this type of structure where the amide nitrogen is directly connected to the fused ring, the five-membered indane fused ring has a better in vitro inhibition effect on *F. equiseti* than the six-membered tetralin fused ring. However, the difference in this structure is only reflected in some of the tested strains, and there is no difference in MIC between *F. solani* and *C. musae*.

Compound **III** does not adopt the mode that the fused ring is directly connected to the amide nitrogen, but it introduces the diphenyl ether structure through the -CH_2_- benzyl group as the connecting arm. This structural modification brought a marked improvement in the inhibitory effect against *F. heterosporum*, with the MIC further reduced to 25 mg/mL. The experimental results show that, compared with the structure of compounds (**I**, **II**) directly bonding nitrogen atoms, compound **III** with a methylene linking arm and a diphenyl ether aryl substitution mode is more conducive to enhancing the in vitro inhibitory effect on some plant pathogenic fungi (*F. equiseti*, *F. heterosporum*, *C. lagenarium*), especially on *F. heterosporum*. It was also found that the same compound had different effects on different fungi, reflecting the strain selectivity of the antifungal activity of this class of enamide derivatives. Therefore, compared with the three tested compounds, compound **III** has the best antifungal performance in vitro.

### 3.6. Preliminary Study on the Antifungal Mechanism of Compound **III**

#### 3.6.1. The Effect of Compound **III** on the Intracellular ROS Level of *F. heterosporum*

**Figure 4:**
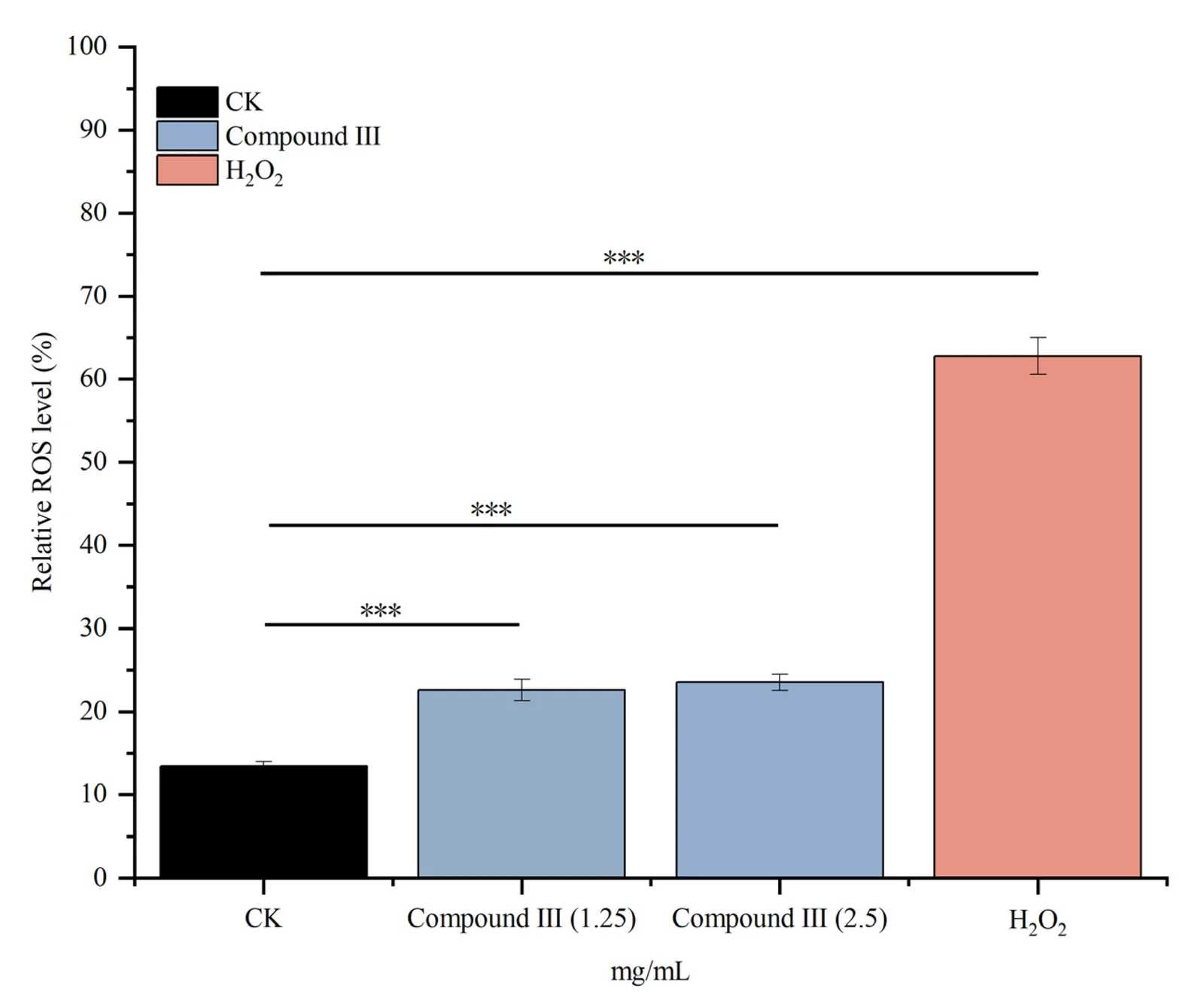
Relative ROS levels under different compound **III** treatments. The data are expressed as the mean ± standard deviation (mean ± SD) of three independent repeated experiments. Compared with the negative control group, the difference in the treatment group reached *** *p* < 0.001. H_2_O_2_ was the positive control for intracellular reactive oxygen species (ROS) production. Compound **III** represented the target compounds treated with 1.25 mg/mL (1/20 MIC) and 2.5 mg/mL (1/10 MIC), respectively.

The outbreak of ROS is one of the most typical early stress responses of eukaryotic cells in response to external drug stress. Excessive ROS can disrupt the cellular redox homeostasis, causing extensive damage to biomolecules [32]. As shown in Figure 4, compound **III** treatment significantly induced the accumulation of intracellular ROS in *F. heterosporum*. The relative ROS level of the negative control group was 13.41%. After treatment with 1.25 mg/mL and 2.5 mg/mL compound **III**, the ROS levels increased to 22.59% (*p* < 0.001) and 23.52% (*p* < 0.001), respectively. The compound treatment group and the H_2_O_2_ positive control group (62.81%) were able to cause an increase in intracellular ROS levels. The results showed that compound **III** treatment could induce the oxidative stress response of *F. heterosporum*.

#### 3.6.2. The Effect of Compound **III** on the MDA Content of *F. heterosporum*

**Figure 5:**
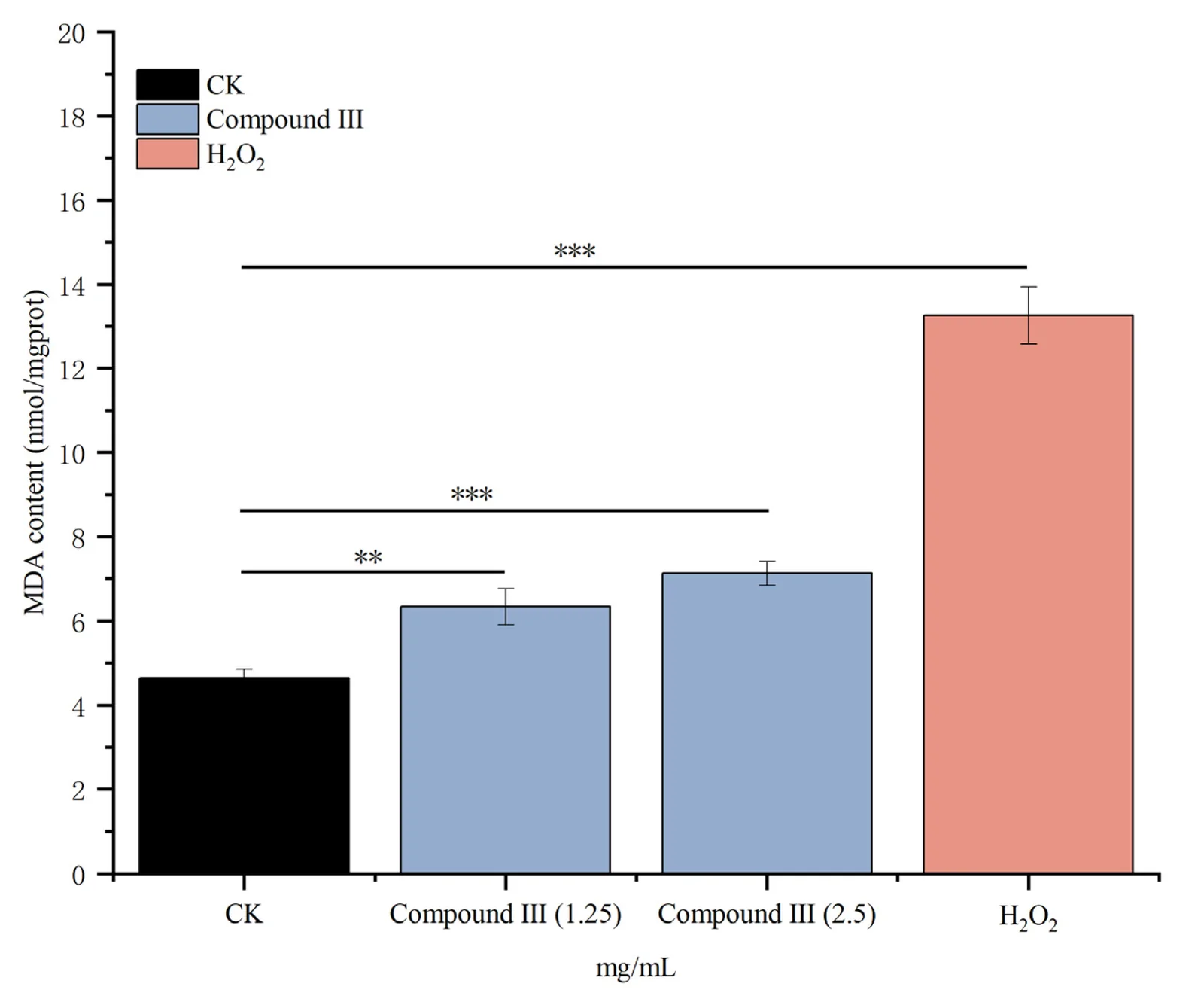
Changes in intracellular MDA content under different compound **III** treatments. The data were expressed mean ± SD of three independent repeated experiments. Compared with the negative control group (CK), ** *p* < 0.01, *** *p* < 0.001. H_2_O_2_ served as the positive control for lipid peroxidation induction. Compound **III** in the figure represents the treatment groups at concentrations of 1.25 mg/mL (1/20 MIC) and 2.5 mg/mL (1/10 MIC), respectively.

MDA is a key marker of lipid peroxidation of cell membrane polyunsaturated fatty acids attacked by ROS. The increase in MDA content indicates lipid peroxidation damage of the fungi [33]. As shown in Figure 5, compound **III** treatment resulted in a significant accumulation of MDA content in fungal cells. The MDA content in the control group was 4.64 nmol/mg prot. After treatment with 1.25 mg/mL and 2.5 mg/mL, the MDA content increased significantly to 6.34 nmol/mg prot (*p* = 0.002) and 7.13 nmol/mg prot (*p* < 0.001), respectively. The MDA content in the H_2_O_2_ positive control group was also significantly increased to 13.26 nmol/mg prot (*p* < 0.001).

#### 3.6.3. The Effect of Compound **III** on the GSH Content of *F. heterosporum*

**Figure 6:**
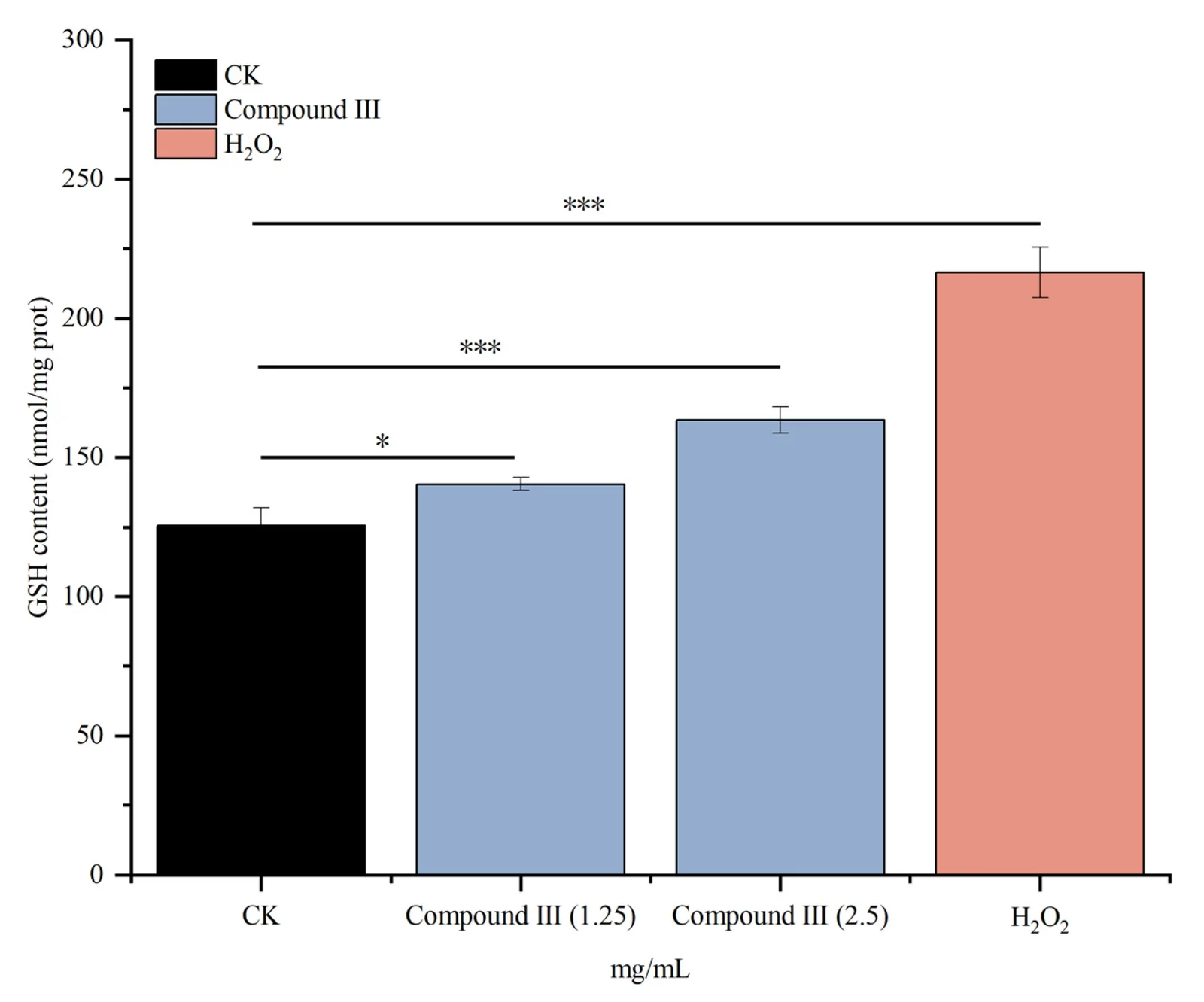
Changes in intracellular GSH content under different compound **III** treatments. The data were expressed as the mean ± SD of three independent repeated experiments. Compared with the negative control group (CK), * *p* < 0.05, *** *p* < 0.001. H_2_O_2_ is a positive control that induces oxidative stress. Compound **III** represented the target compounds treated with 1.25 mg/mL (1/20 MIC) and 2.5 mg/mL (1/10 MIC), respectively.

GSH is the core non-enzymatic antioxidant in cells, and the fluctuation of its content directly reflects the resistance and compensation ability of cells to oxidative stress [34]. As shown in Figure 6, after treatment with compound **III**, the intracellular GSH content of fungi was significantly higher than that of the control group. Compared with the control group (125.58 nmol/mg prot), the GSH levels of 1.25 mg/mL and 2.5 mg/mL treatment groups increased by 11.8% (140.44 nmol/mg prot, *p* < 0.05) and 30.1% (163.44 nmol/mg prot, *p* < 0.001), respectively. The GSH level in the H_2_O_2_ positive control group increased by 72.3% (216.44 nmol/mg prot, *p* < 0.001).

#### 3.6.4. The Effect of Compound **III** on the Cell Membrane Integrity of *F. heterosporum*

**Figure 7:**
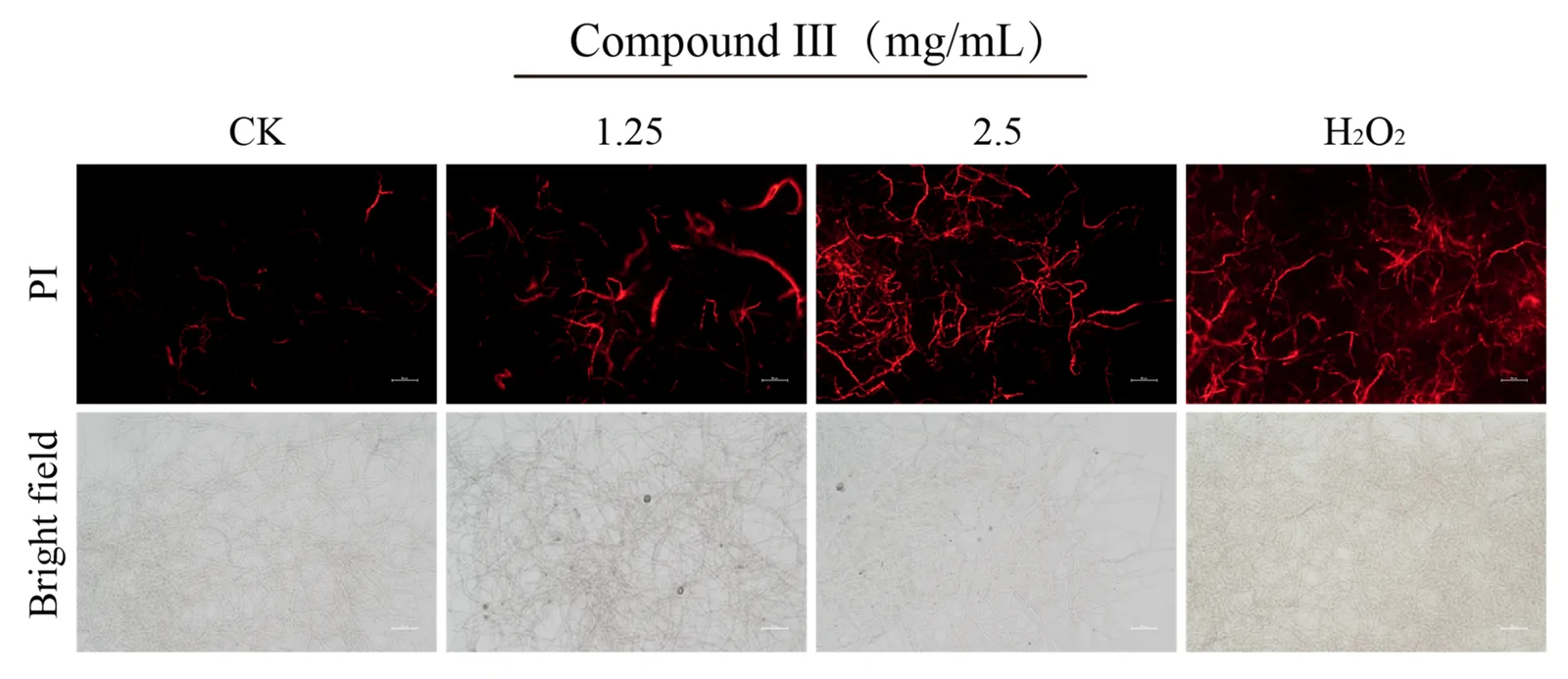
Evaluation of cell membrane integrity by propidium iodide staining.

This study used PI staining to detect the effect of compound **III** on fungal cell membrane integrity. As a nucleic acid dye, PI cannot penetrate intact cell membranes, but it can enter cells with damaged membrane structures and produce red fluorescence [35]. As shown in Figure 7, the negative control group (CK) of *F. heterosporum* mycelium showed only a very weak background fluorescence signal. In contrast, hyphae treated with compound **III** at 1.25 mg/mL and 2.5 mg/mL exhibited obvious red fluorescence, and the red fluorescence signal in the 2.5 mg/mL-treated group was stronger than that in the 1.25 mg/mL-treated group. These results indicate that compound **III** can induce cell membrane damage in *F. heterosporum*.

Hyphae were stained with PI after treatment with different concentrations of compound **III** (1.25 and 2.5 mg/mL) and positive control (H_2_O_2_), and they were then observed under a fluorescence microscope. All experiments were performed in triplicate. The first row shows the fluorescence channel, and the second row shows the bright-field channel; scale bar = 50 μm.

### 3.7. Compound **III** Exposure Affects Embryonic Development in Zebrafish Larvae

**Figure 8:**
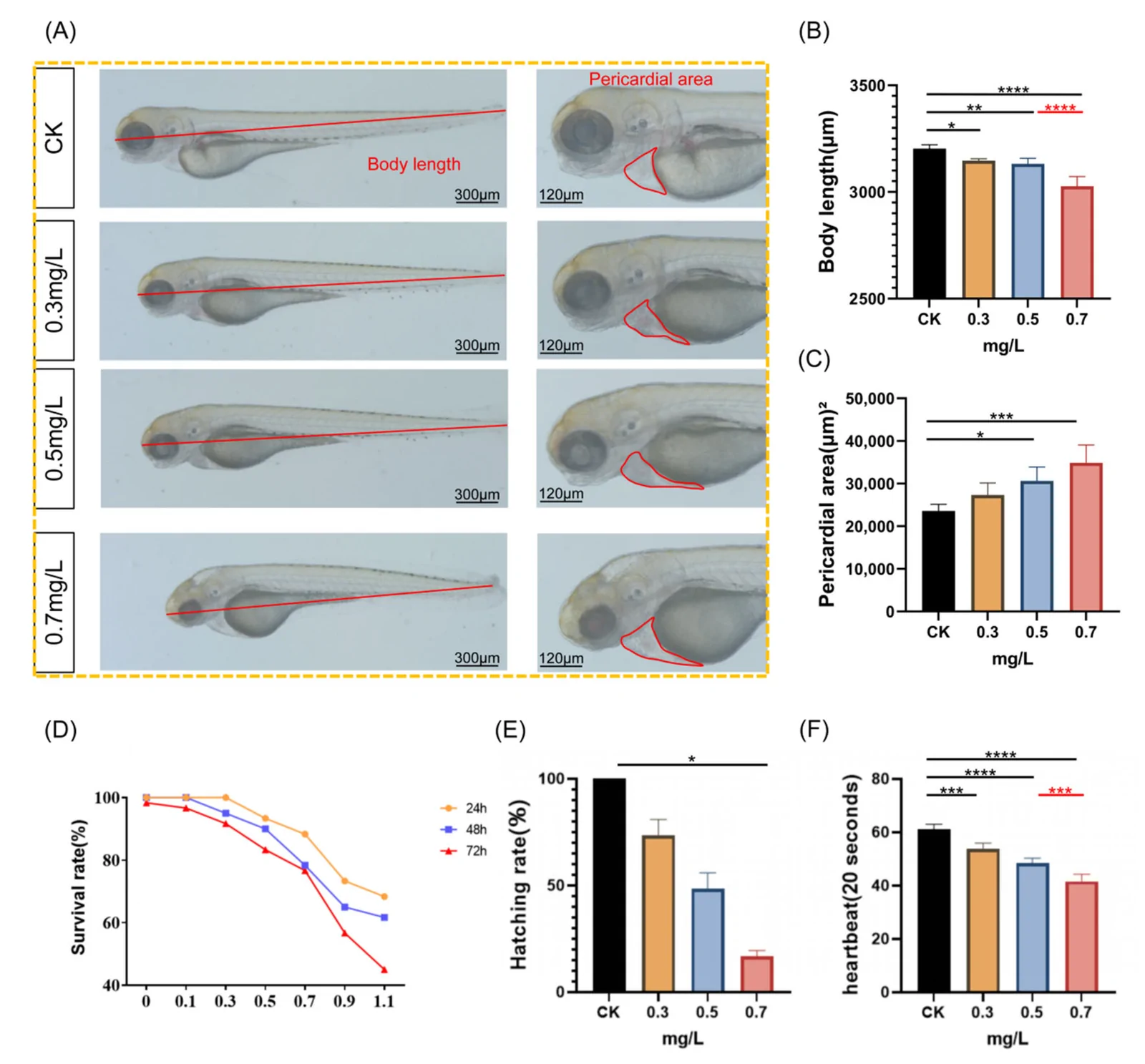
Effects of compound **III** exposure on the growth and development of zebrafish embryos. (**A**) A total of 72 hpf representative bright-field images of zebrafish larvae in each treatment group. The left image shows the overall shape (red line segments indicate body length, scale: 300 μm). The image on the right is an enlarged view of the pericardial area (red outline indicating pericardial area, scale: 120 μm). (**B**) Body length statistics at 72 hpf *n* = 5. (**C**) Statistics of pericardial area of zebrafish embryos at 72 hpf *n* = 5. (**D**) Survival rates of zebrafish embryos at 24, 48, and 72 hpf under exposure to different concentrations of compound **III** *n* = 3. (**E**) At 72 hpf, the hatching rate of zebrafish embryos was *n* = 3. (**F**) At 72 hpf, the number of heartbeats per 20 s of zebrafish embryos was *n* = 5. The data are expressed as the mean ± SD. One way ANOVA and Tukey’s post hoc test were used for intergroup comparison. The difference is considered statistically significant with *p* < 0.05. The significance markers are * *p* < 0.05, ** *p* < 0.01, *** *p* < 0.001, **** *p* < 0.0001. Black asterisks indicate comparisons with the control group (CK), while red asterisks indicate pairwise comparisons between dose groups.

In order to evaluate the developmental toxicity of compound **III** on zebrafish embryos, this study investigated its dose-effect and time-effect relationship. As shown in Figure 8D, the survival rate of embryos decreases in a dependent manner with an increase in exposure concentration and time; The median lethal concentration (LC_50_) of 72 hpf was fitted using a non-linear regression (log–logistic model) and found to be 1.032 mg/L (95% CI: 0.975–1.111). At sublethal concentrations, the body length of juvenile fish in the treatment group showed a concentration-dependent shortening (Figure 8A,B). The body length of the 0.7 mg/L group was 3027 ± 45 μm, which was about 5.5% shorter than the control group (3203 ± 18 μm) (*p* < 0.0001, Tukey). The hatching rate decreased from 100% in the control group to 16.7% ± 2.9% in the 0.7 mg/L group (Figure 8E). In addition, the pericardial area of the treatment group showed a concentration-dependent increase (Figure 8A,C), reaching 34,910 ± 4134 μm^2^ in the 0.7 mg/L group, which was approximately 47.8% higher than the control group (23,615 ± 1544 μm^2^) (*p* < 0.001). The heart rate decreased from 61.0 ± 2.0 beats/20 s in the control group to 41.6 ± 2.7 beats/20 s in the 0.7 mg/L group (Figure 8F), a decrease of about 31.8% (*p* < 0.0001), showing pericardial edema and impaired cardiac function. In summary, compound **III** exhibits concentration-and time-dependent developmental toxicity to zebrafish embryos, manifested as lethal effects, growth inhibition, and cardiac toxicity. These phenotypes suggest that it may have neurotoxicity, which will be further validated through behavioral testing and Tg (elavl3: EGFP) transgenic fish lines.

### 3.8. Effects of Compound **III** on the Neurodevelopment and Locomotor Behavior of Zebrafish Larvae

**Figure 9:**
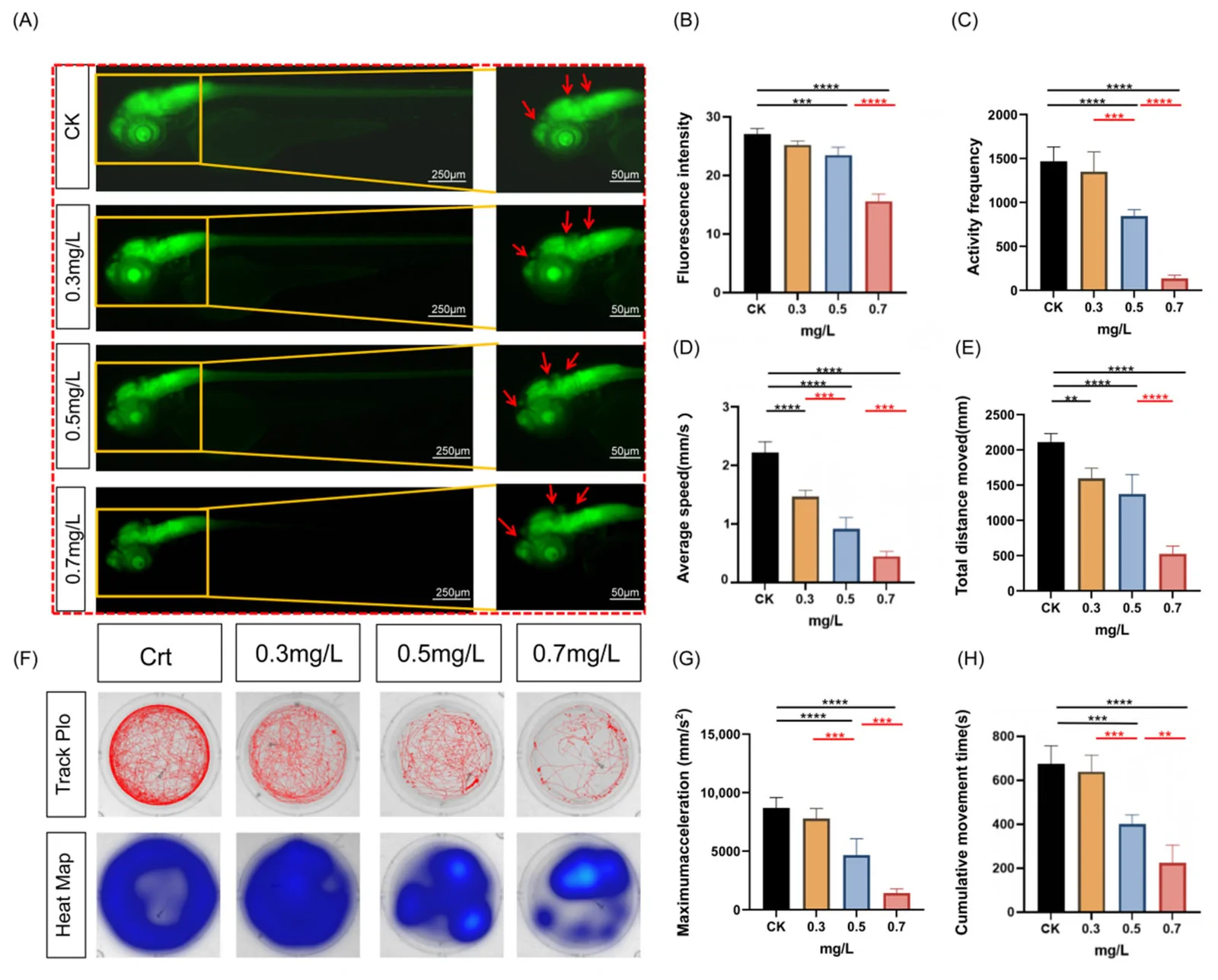
Effects of compound **III** exposure on neuronal development and spontaneous motor behavior in the head of zebrafish larvae. (**A**) Representative fluorescence images of head neurons in each treatment group. The left side is listed as a holistic view (scale: 250 μm). The right column is a local enlarged image (scale: 50 μm), and the arrow indicates the area where the neuron is missing (the fluorescence signal is weakened). (**B**) Quantitative analysis of fluorescence intensity of head neurons *n* = 5. (**C**–**H**) Detection of spontaneous motor behavior parameters: (**C**) activity frequency *n* = 5; (**D**) average speed *n* = 5; (**E**) total movement distance *n* = 5; (**F**) motion trajectory diagram (top) and heatmap (bottom); (**G**) maximum acceleration *n* = 5; (**H**) accumulated exercise time *n* = 5. The data were expressed as the mean ± SD. One-way ANOVA and Tukey’s post hoc test were used for comparison between groups. The difference is considered statistically significant with *p* < 0.05. The significance markers are ** *p* < 0.01, *** *p* < 0.001, **** *p* < 0.0001. Black asterisks indicate comparisons with the control group (CK), while red asterisks indicate pairwise comparisons between dose groups.

In order to further explore the toxic effects of compound **III** on the nervous system of zebrafish, the morphology and spontaneous motor behavior of head neurons in juvenile fish were observed at sublethal concentrations. As shown in Figure 9A,B, compared with the control group, the fluorescence intensity of neurons in the head of juvenile fish in the compound **III** treatment group showed a concentration-dependent decrease. The fluorescence intensity of the 0.7 mg/L group was 15.6 ± 1.2, which was about 42.2% lower than the control group (27.0 ± 1.0) (*p* < 0.0001, Tukey). The neurons in the high concentration group were sparsely arranged and the fluorescence signal was significantly weakened, suggesting that the compound can interfere with the development or survival of neurons. The results of behavioral tests (Figure 9C–H) showed that the exercise parameters of the juvenile fish in the treatment group were decreased in a concentration-dependent manner compared with the control group. The average exercise speed decreased from 2.22 ± 0.18 mm/s in the control group to 0.45 ± 0.09 mm/s in the 0.7 mg/L group (a decrease of approximately 79.9%, *p* < 0.0001). The total movement distance decreased from 2108 ± 125 mm to 527 ± 111 mm (a decrease of approximately 75.0%, *p*< 0.0001). The activity frequency decreased from 1467 ± 165 to 136 ± 36 (a decrease of approximately 90.7%, *p* < 0.0001), and the maximum acceleration decreased from 8691 ± 891 mm/s^2^ to 1435 ± 342 mm/s^2^ (a decrease of approximately 83.5%), *p* < 0.0001). The cumulative exercise time decreased from 674 ± 83 s to 224 ± 80 s (a decrease of approximately 66.7%, *p* < 0.0001). It was found that only the average movement speed and total movement distance decreased significantly at the lowest concentration of 0.3 mg/L, while the activity frequency, maximum acceleration, and cumulative movement time did not change significantly, suggesting that movement speed and movement distance were more sensitive early behavioral indicators. The movement trajectory and heat map further showed that the movement range of the juvenile fish in the high concentration group was significantly reduced, and the activity hotspots were significantly reduced, showing slow movement and inhibition of exploratory behavior (Figure 9F). In conclusion, compound **III** exposure can cause neuronal structural damage and motor dysfunction in zebrafish larvae, indicating that it has significant neurotoxicity; combined with the above developmental toxicity and cardiotoxicity, it is suggested that compound **III** has a multi-system toxic effect on zebrafish embryos.

## 4. Discussion

The crystal structure analysis shows that the change of substituents can significantly regulate the crystal system, space group, and supramolecular stacking mode of these derivatives: compounds **I**–**III** form dimer stacking, herringbone, and layered stacking structures, respectively. The strong N-H···O hydrogen bond between molecules is the dominant driving force for stabilizing the crystal phase [36], and the weak interactions such as C-H···O and C-H···π play a key synergistic role. The π-π stacking only exists in some systems. Even in the absence of strong π-π interactions, these flexible molecules can still construct highly stable three-dimensional supramolecular networks and achieve ordered crystallization under the joint dominance of strong hydrogen bonds and multiple weak supramolecular interactions. The above results may be understood to provide direct structural evidence for the solid-state stability of this class of N-substituted-3-methylbut-2-enamide derivatives.

In the present study, compounds **I** and **II** were prepared by an acyl chloride/triethylamine system. The amino group of the amine substrate used for compound **I** is located at the C1 position of the tetrahydronaphthalene ring. This site is relatively sterically crowded, which may reduce the nucleophilic attack ability of the primary amine toward the acyl chloride, thereby decreasing the amidation efficiency. In addition, acyl chloride is prone to hydrolysis, trace water in the reaction system may consume acyl chloride raw materials, and the column chromatography purification process may also cause product loss. It should be noted that this study did not set up a control experiment to quantitatively evaluate the effect of acyl chloride hydrolysis on the yield, nor did it systematically optimize the reaction conditions for the high-resistance substrate. Under the same acyl chloride/triethylamine reaction conditions, compound **II** was obtained from 1-aminoindan with a separation yield of 43.3%. The isolated yield of compound **I** was 25.3%. From the comparison of the substrate skeletons of the two, the steric hindrance of the indane ring to the C1 amino group is smaller than that of the tetrahydronaphthalene ring. Combined with the synthesis results of compounds **I** and **II**, it can be inferred that the steric hindrance of the substrate is the main factor for the low separation yield of compound **I**, but this inference still needs to be verified by controlled experiments and condition optimization. As far as the amidation strategy is concerned, two methods were used in this study. The acyl chloride/triethylamine method proceeds rapidly with inexpensive acylating reagents, but strict anhydrous conditions are mandatory because acyl chlorides readily undergo hydrolysis. In our experiments, this approach gave low conversion for sterically hindered C1-benzyl primary amine substrates, consistent with the well-known difficulty of acylation for sterically encumbered amines [37]. Therefore, compound **III** was synthesized using the EDCI/DMAP condensation protocol. Relative to DCC-type reagents, EDCI has a practical advantage: its urea byproduct (EDU) is water-soluble and can be eliminated during aqueous workup [38]. DMAP serves as an acyl transfer catalyst for this EDCI-mediated coupling [39]. We did not attempt the EDCI/DMAP protocol for the synthesis of compound **I** in this work, and whether this method is applicable to this substrate remains to be explored in further studies.

In this study, 3-methyl-2-butenamide was used as the parent nucleus, and different skeleton types of substituents (tetrahydronaphthalene-1-yl, dihydroinden-1-yl, and 4-phenoxybenzyl) were introduced into the amide nitrogen atom to preliminarily explore the effect of amide N-position substituent structure on antifungal activities. The design aims to perform preliminary skeleton screening rather than systematic quantitative SAR analysis. The MIC of compound **I** against the six tested fungi is 100 mg/mL, which is one of the compounds with weak activity in the synthetic target compound. The six-membered tetralin fused ring in compound **I** was replaced by a smaller five-membered indane skeleton to obtain compound **II**. It has been confirmed that the steric hindrance of the indane skeleton is significantly smaller than that of the tetrahydronaphthalene structure. This structural modification reduces the overall rigidity of the molecule of compound **II** and reduces the free volume of the ring system [40]. Compared with compound **I**, the MIC of compound **II** against *F. equiseti* was decreased from 100 mg/mL to 50 mg/mL, and the antifungal activity was doubled. It is speculated that the change of spatial effect caused by moderate ring contraction may have a beneficial effect on its activity. Compound **III** introduces the diphenyl ether structure into the N-terminus of the amide through the methylene linking arm (-CH_2_-). Compared with the rigid compounds **I** and **II** (MIC is 100 mg/mL) directly connected to the fused ring, the MIC of compound **III** against *F. heterosporum* decreased to 25 mg/mL, and the antifungal activity increased by three times. It is speculated that appropriately enhanced molecular flexibility and larger aromatic surface area could favor the antifungal potency of this series of enamide derivatives. For a preliminary assessment of the practical application potential of compound **III**, its antifungal activity was qualitatively compared with the MIC values of carbendazim against related phytopathogenic fungi reported in the literature. In that study, the MIC values of carbendazim against *Fusarium oxysporum*, *F. solani*, and *Fusarium graminearum* were 1.95 μg/mL, 7.8 μg/mL, and 3.9 μg/mL, respectively [41]. However, it should be noted that this comparison is only indirect, because the MIC values reported in different studies may vary considerably depending on inoculation concentration, culture medium, and culture conditions. However, only three target compounds were selected in this study, and the structural diversity was limited, which was not enough to systematically elucidate the regulation of different substituents on antifungal activity. In addition, the MIC values of all compounds are at a high level, and there is still much room for improvement in activity. In the future, it is necessary to further expand the scope of structural modification in order to screen out more active enamide lead compounds.

Oxidative stress is one of the important dimensions to evaluate the mechanism of antifungal compounds [42]. After treatment with compound **III**, the intracellular ROS level of *F. heterosporum* increased from 13.41% in the control group to 22.59% (1.25 mg/mL) and 23.52% (2.5 mg/mL), respectively. The GSH content increased from 125.58 nmol/mg prot in the control group to 140.44 nmol/mg prot (1.25 mg/mL) and 163.44 nmol/mg prot (2.5 mg/mL), respectively. The MDA content increased from 4.64 nmol/mg prot in the control group to 6.34 nmol/mg prot (1.25 mg/mL) and 7.13 nmol/mg prot (2.5 mg/mL), respectively. Comparing the compound **III** high concentration group with the H_2_O_2_ positive control group, the ROS level of the H_2_O_2_ group (62.81%) was about 2.7 times higher than that of the compound **III** high concentration group, and its GSH increase (72.3%) was about 2.4 times higher than that of the latter, while the MDA increase (185.8%) was about 3.5 times higher than that of the latter. The comparison was only based on two concentration points and one positive control, which was not enough to establish a strict dose-effect equation. The bispyrazole amide derivative B8, which is similar to the structure type of this compound, can induce ROS accumulation and lipid peroxidation damage after treatment with *Sclerotinia sclerotiorum* [43]. The pyrazole-5-sulfonamide derivative C22 also exerts antifungal effects by inducing oxidative damage and destroying cell membrane integrity [44]. The above literature did not report any specific changes in GSH and could not be directly compared with the GSH data of this experiment. Based on the above results, these findings suggest that the antifungal activity of compound **III** may be related to the induction of oxidative stress and the destruction of fungal cell membrane integrity. Nevertheless, the existing data cannot confirm whether oxidative stress is a direct cause of cell membrane damage, nor can it be determined whether the two are events that occur independently of each other after treatment with the compound. Further experiments including GSH/GSSG ratio detection are needed to analyze the internal relationship between the two.

In this study, compound **III** was observed to induce concentration-and time-dependent developmental toxicity in zebrafish embryos, manifested as lethality, growth inhibition, and cardiac toxicity phenotypes such as pericardial edema and bradycardia. Based on these findings, we further performed fluorescence microscopy to observe neuronal fluorescence intensity in Tg(elavl3:EGFP) zebrafish and conducted behavioral tests. At sublethal concentrations, reduced neuronal fluorescence signals in the larval head and impaired motor function were observed. At the lowest tested concentration, only swimming velocity and total travel distance decreased significantly, suggesting that these two endpoints may serve as sensitive early biomarkers, with this hypothesis requiring further validation. Taken together, these results indicate that compound **III** exerts multi-system toxicity in zebrafish embryos. The concurrent occurrence of cardiac and neurotoxic phenotypes raises the possibility that these phenotypes share upstream processes or that one may be secondary to the other; however, pathway validation was not performed in this study. Structurally, Compound **III** is an *α*,*β*-unsaturated amide, possessing the same *α*,*β*-unsaturated carbonyl scaffold as acrylamide. Its 4-phenoxybenzyl fragment bears similarity to the pharmacophore of pyrethroids. This study did not perform a side-by-side comparison of the toxicity, including LC_50_ values, of different compounds. Acrylamide and pyrethroids belong to different chemical classes, and their mechanisms of toxicity differ fundamentally. The relevant literature was used only to interpret potential toxicity clues associated with different structural fragments of the target molecule; it serves only as an indirect reference for structural toxicity inference and should not be directly extrapolated to the compounds in this study. The previous literature has reported that acrylamide induces reduced locomotor activity and neuronal morphological alterations in zebrafish. The *β*-carbon of its *α*,*β*-unsaturated amide conjugated system serves as a soft electrophilic center and can undergo Michael addition with the reduced thiol groups of cysteine residues in proteins under physiological conditions to form covalent adducts [45]. Zebrafish embryo studies have further shown that the electrophilicity and hydrophobicity of *α*,*β*-unsaturated amide compounds can regulate their toxic effects [46]. For pyrethroid compounds, previous zebrafish studies have shown that cardiac toxicity phenotypes are not consistent across different agents: deltamethrin caused a 15.75% decrease in heart rate in the F1 generation and induced bradycardia, whereas fenpropathrin produced a dose-dependent increase in heart rate [47,48]. Based on the aforementioned structure–toxicity relationships reported in the literature, we speculate that the multi-system toxicity of compound **III** may be associated with the *α*,*β*-unsaturated amide scaffold and 4-phenoxybenzyl substituent. However, physicochemical parameters of the target compound were not determined in the present study. Therefore, this structure–toxicity association remains a hypothesis awaiting validation. This study was based solely on phenotypic observations of a single compound, lacking analog comparison and pathway validation. Therefore, it is insufficient to establish SARs, and the precise mechanism remains to be confirmed. The causal relationships among neuronal injury, cardiac abnormalities and developmental retardation remain unclear. Neuronal and behavioral impairments may be secondary effects resulting from global developmental delay or cardiac dysfunction, rather than specific neurotoxicity. This phenotypic combination is suggestive for developmental toxicant screening, yet risk extrapolation still requires validation. Further studies incorporating transcriptomics, neurotransmitter quantification, and analog comparison will help to dissect its molecular mechanism.

The antifungal activity was determined by the filter paper diffusion method. The MIC (25–100 mg/mL) reported in this paper was the concentration of mother liquor impregnated with filter paper, which was not the final concentration of actual contact of fungi in agar medium. In the filter paper disk diffusion system, the compound gradually diffuses from the filter paper disk into the agar matrix. The actual exposure concentration at the fungal growth site is markedly lower than the impregnating stock solution concentration. Therefore, this stock solution concentration should not be directly compared with the 72 hpf LC_50_ (1.032 mg/L) obtained from the semi-static aqueous immersion exposure of zebrafish embryos, nor can a safety factor be directly calculated based on these two sets of values. Although the exposure media and exposure methods of the two experiments were different, the zebrafish embryo toxicity results suggested that compound **III** had potential aquatic biological risks. The compound has in vitro antifungal activity against the tested plant pathogens, but it shows strong aquatic toxicity to zebrafish embryos, which limits its practical application in agriculture. Overall, although this compound possesses potential for antifungal development, its aquatic safety risk cannot be ignored. Future studies should therefore focus on molecular structural modification to balance antifungal activity and aquatic toxicity, thereby providing a reference for the screening of lead molecules for novel green fungicides.

## 5. Conclusions

This study obtained three N-substituted-3-methylbut-2-enamide derivatives and completed structural characterization. Antifungal activity tests showed that the diphenyl ether-substituted enamide compound **III** bearing a methylene flexible linker exhibited the optimal activity against *F. heterosporum*. This compound is capable of inducing oxidative stress in fungi and impairing cell membrane integrity, thereby exerting antifungal activity. Zebrafish embryo toxicity experiments showed that compound **III** had a concentration-and time-dependent developmental toxicity to zebrafish embryos. Under the 72 hpf exposure condition, the LC_50_ was 1.032 mg/L. At sublethal exposure concentrations, the compound can reduce embryo survival, lead to developmental delay, and induce neurobehavioral abnormalities. Compound **III** exhibits in vitro antifungal activity against the tested plant pathogenic fungi, yet it shows high aquatic toxicity to zebrafish embryos, which limits its practical application in agriculture. This result suggests that although compound **III** possesses potential for antifungal development, its aquatic safety risk cannot be ignored. Further molecular structural modification is required to balance antifungal activity and aquatic toxicity, providing a reference for the screening of lead molecules of novel green fungicides.

## Figures and Tables

**Table 1:** Crystal data and structural parameters of the three compounds.

Parameters	Compounds		
	Compound I	Compound II	Compound III
Empirical formula	C_15_H_19_NO	C_14_H_17_NO	C_18_H_19_NO_2_
Formula weight	229.31	215.28	280.33
Temperature/K	150.15	120.00(10)	150.00(10)
Crystal system	triclinic	monoclinic	orthorhombic
Space group	P1	P21/c	P212121
*a*/Å	8.9086(4)	11.6805(3)	5.47422(7)
*b*/Å	8.9141(5)	11.3228(2)	9.58669(12)
*c*/Å	18.5488(8)	9.7294(2)	29.2280(4)
*α*/°	79.773(4)	90	90
*β*/°	87.487(4)	114.000(3)	90
*γ*/°	64.076(5)	90	90
*V*/Å^3^	1302.80(12)	1175.53(5)	1533.87(3)
*Z*	4	4	4
ρcalcg/cm^3^	1.169	1.216	1.214
*μ*/mm^−1^	0.564	0.594	0.629
*F*(000)	496	464	596
Crystal size/mm^3^	0.06 × 0.06 × 0.05	0.15 × 0.12 × 0.1	0.16 × 0.14 × 0.12
Radiation	CuKα (*λ* = 1.54184)	CuKα (*λ* = 1.54184)	CuKα (*λ* = 1.54184)
2θ range for data	9.698 to 146.636	8.286 to 145.876	9.71 to 145.852
collection/°			
Index ranges	−10 ≤ h ≤ 10, −10 ≤ k ≤ 10, −22 ≤ l ≤ 22	−14 ≤ h ≤ 14, −14 ≤ k ≤ 13, −8 ≤ l ≤ 11	−6 ≤ h ≤ 6, −11 ≤ k ≤ 8, −34 ≤ l ≤ 35
Reflections collected	7137	12,568	10,124
Independent reflections	7137 [Rint = 0.0658, Rsigma = 0.0544]	2201 [Rint = 0.0132, Rsigma = 0.0081]	2956 [Rint = 0.0157, Rsigma = 0.0125]

**Table 2:** Determination of minimum inhibitory concentrations (MICs) of the three compounds against six fungi.

Strain	Compounds	Concentration (mg/mL)						
		100	50	25	12.5	6.25	Negative Control	Positive Control
*F. solani*	**I**	−	+	+	+	+	+	−
	**II**	−	+	+	+	+	+	−
	**III**	−	+	+	+	+	+	−
*C. musae*	**I**	−	+	+	+	+	+	−
	**II**	−	+	+	+	+	+	−
	**III**	−	+	+	+	+	+	−
*F. equiseti*	**I**	−	+	+	+	+	+	−
	**II**	−	−	+	+	+	+	−
	**III**	−	−	+	+	+	+	−
*F. heterosporum*	**I**	−	+	+	+	+	+	−
	**II**	−	+	+	+	+	+	−
	**III**	−	−	−	+	+	+	−
*M. fructigena*	**I**	−	+	+	+	+	+	−
	**II**	−	+	+	+	+	+	−
	**III**	−	+	+	+	+	+	−
*C. lagenarium*	**I**	−	+	+	+	+	+	−
	**II**	−	+	+	+	+	+	−
	**III**	−	−	+	+	+	+	−

Note: “+” indicates mycelial growth under the filter paper strip at that concentration, while “−” indicates no mycelial growth under the filter paper strip at that concentration.

## Data Availability

All data supporting the findings of this study are included in the article and its Supplementary Materials.

## Supplementary Materials

The following supporting information can be downloaded at https://www.mdpi.com/article/10.3390/biology15212003/s1: File S1. NMR spectra; File S2. Crystal data. Crystallographic data of compound **I** (3-methyl-N-(1,2,3,4-tetrahydronaphthalen-1-yl)but-2-enamide); compound **II** (N-(2,3-Dihydro-1H-inden-1-yl)-3-methylbut-2-enamide) and compound **III** (3-Methyl-N-(4-phenoxy benzyl)but-2-enamide) have been deposited in the Cambridge Crystallographic Data Centre: Deposition Number(s) CCDC 2536305 (for compound **I**), CCDC 2536306 (for compound **II**), and CCDC 2536307 (for compound **III**), which contain the supplementary crystallographic data for this paper. These data are provided free of charge by the joint Cambridge Crystallographic Data Centre and Fachinformations zentrum Karlsruhe Access Structures service.

## Abbreviations

ANOVA	analysis of variance
BCA	bicinchoninic acid
DMSO	dimethyl sulfoxide
DMAP	4-(dimethylamino)pyridine
EDCI	1-(3-dimethylaminopropyl)-3-ethylcarbodiimide hydrochloride
EGFP	enhanced green fluorescent protein
FET	fish embryo acute toxicity
GSH	reduced glutathione
hpf	hours post-fertilization
LC_50_	lethal concentration 50
MDA	malondialdehyde
MIC	minimum inhibitory concentration
OECD	Organization for Economic Co-operation and Development
PBS	phosphate-buffered saline
PDA	potato dextrose agar
PDB	potato dextrose broth
PI	propidium iodide
PTU	1-phenyl-2-thiourea
ROS	reactive oxygen species
RSD	relative standard deviation
SD	standard deviation
SAR	structure–activity relationship
