# Supplementary material for: Discovery of Bioactive N-Substituted-3-methylbut-2-enamides: Synthesis, Crystal Structures, Antifungal Activity, Mechanism of Action, and Zebrafish Toxicity Evaluation
Source: Biology (Basel). 2026 Nov 4;15(21):2003. doi: 10.3390/biology15212003 (PMC13761460; doi:10.3390/biology15212003)
Supplement: Supplementary file 1 [file biology-15-02003-s001.zip › Replace the originally submitted Supplementary Materials/File S1.pdf]

Spectra of compounds

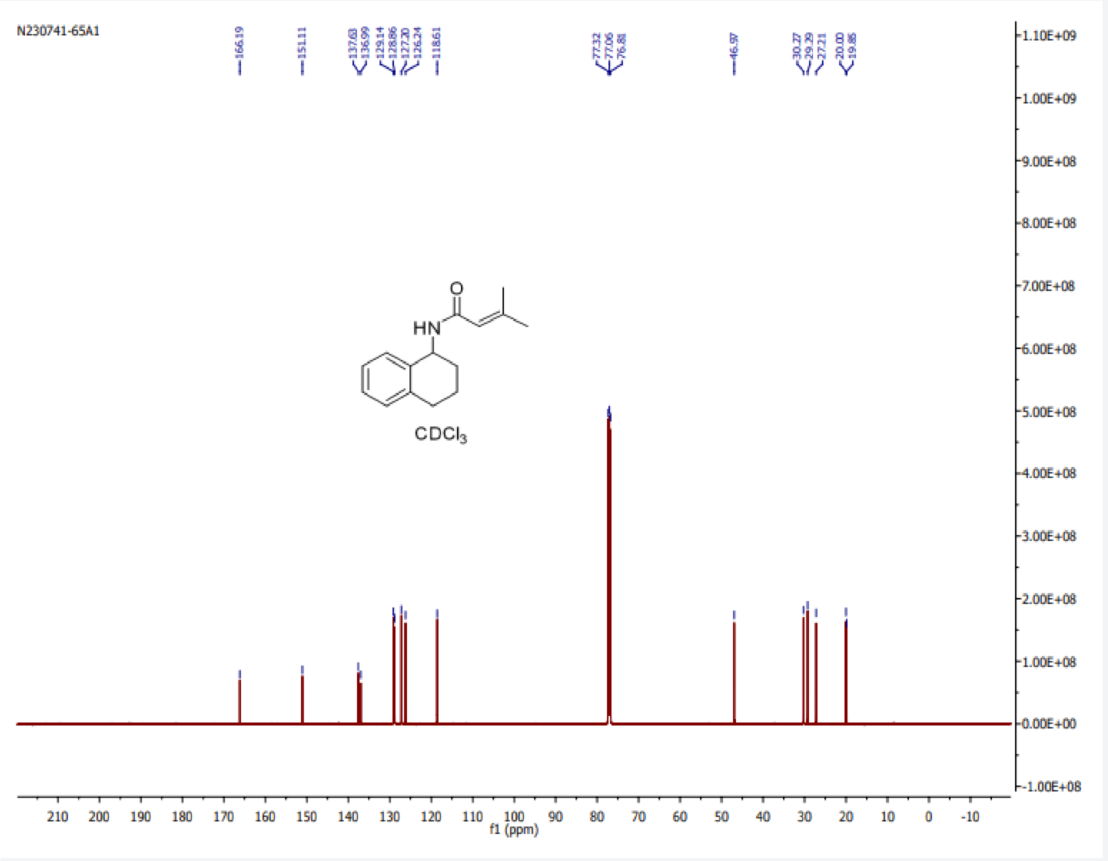

^{13}C NMR spectral data of compound I

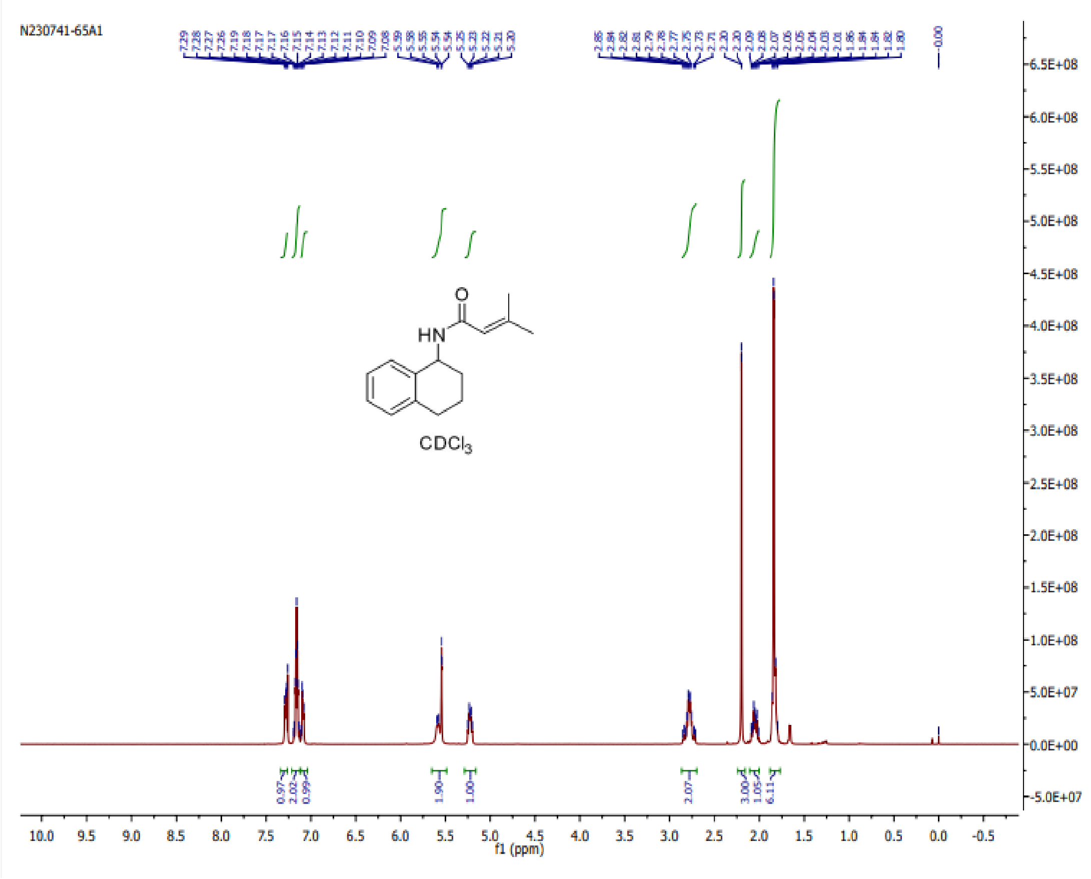

^{1}H NMR spectral data of Compound I

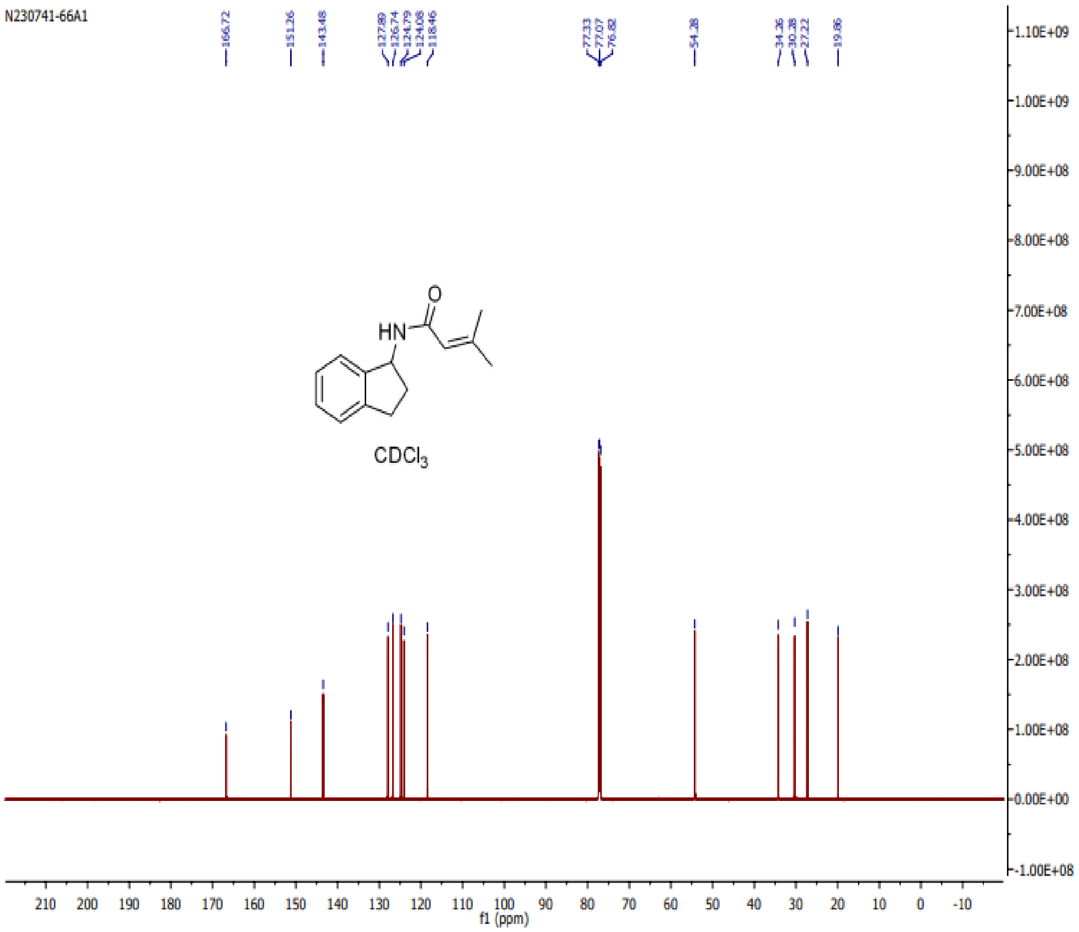

^{13}C NMR spectral data of compound II

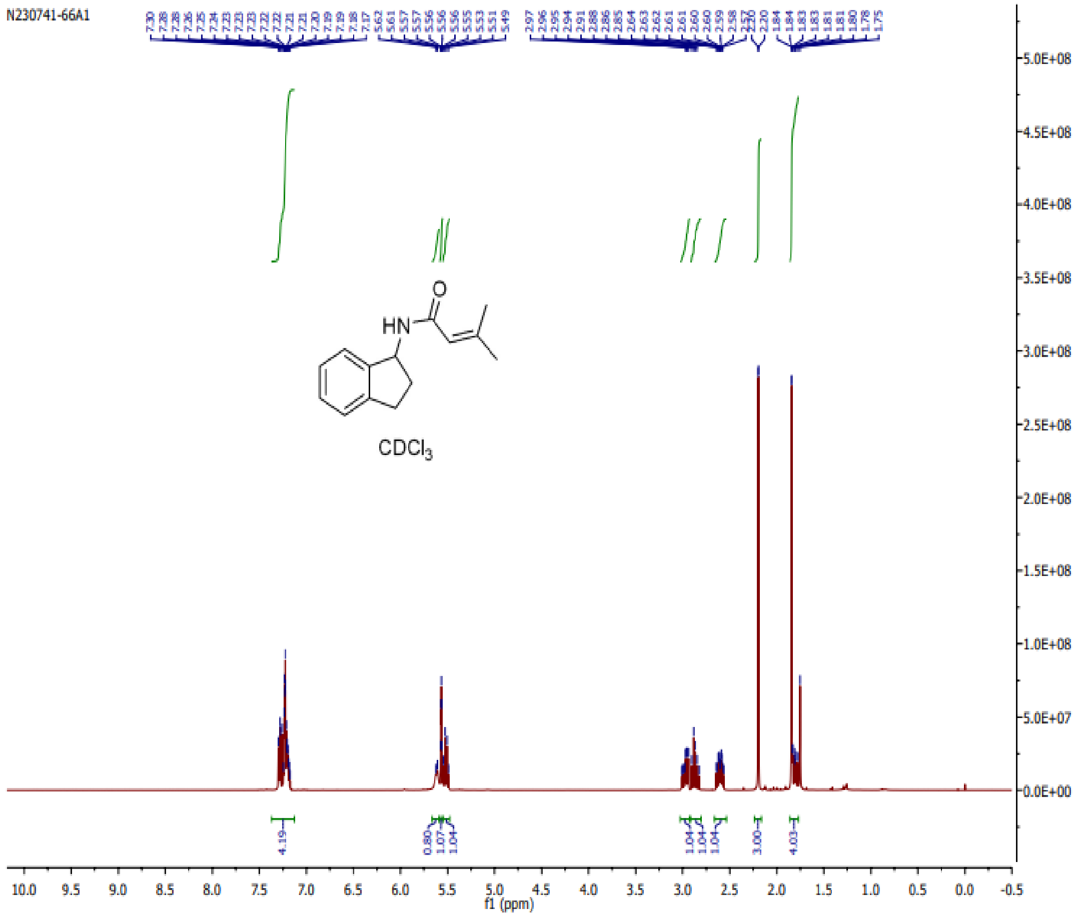

^{1}H NMR spectral data of compound II

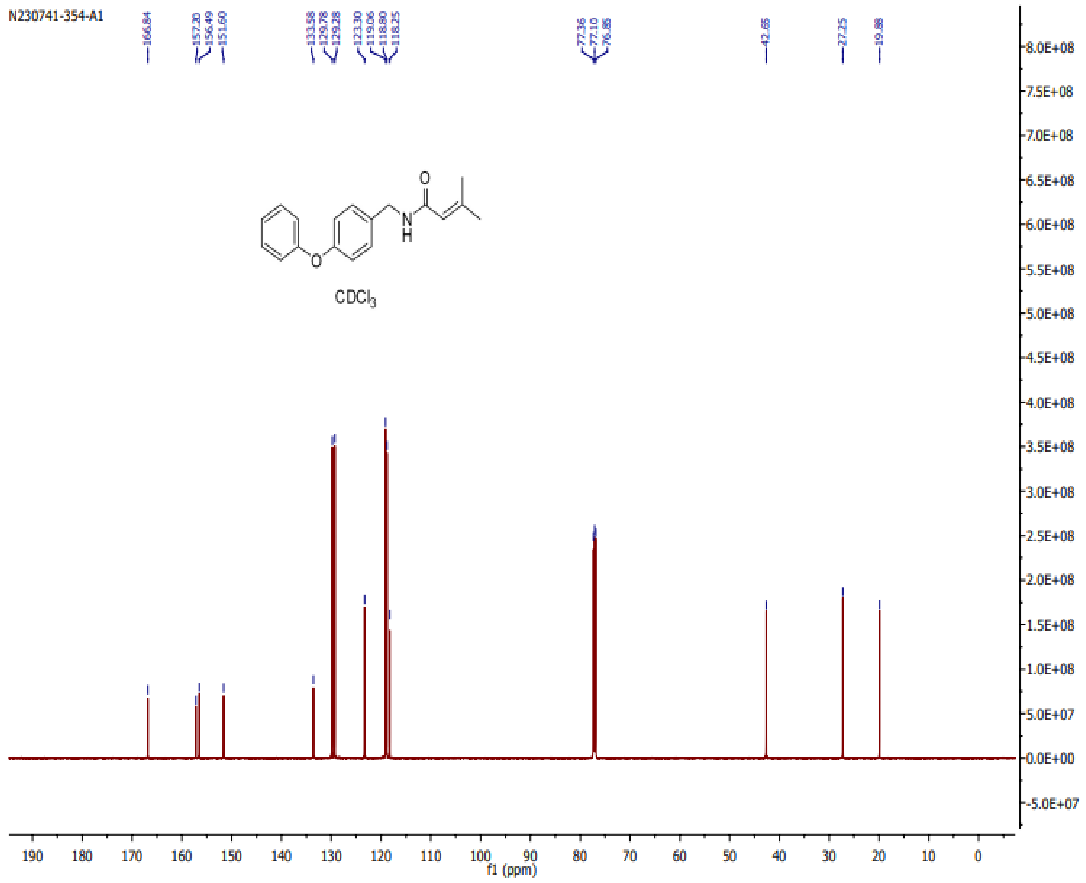

^{13}C NMR spectral data of compound III

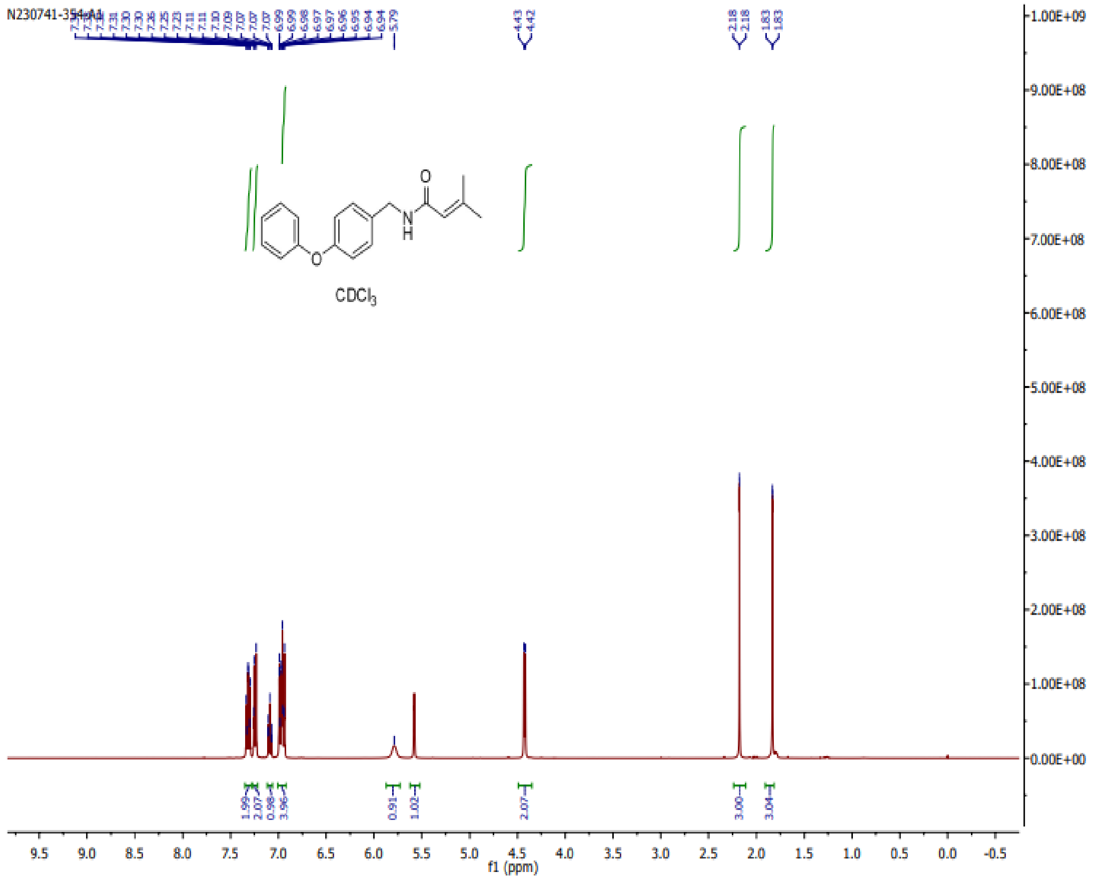

^{1}H NMR spectral data of compound III
