# Supplementary material for: Discovery of Bioactive N-Substituted-3-methylbut-2-enamides: Synthesis, Crystal Structures, Antifungal Activity, Mechanism of Action, and Zebrafish Toxicity Evaluation
Source: Biology (Basel). 2026 Nov 4;15(21):2003. doi: 10.3390/biology15212003 (PMC13761460; doi:10.3390/biology15212003)

checkCIF/PLATON report

Structure factors have been supplied for datablock(s) zhou_xh_auto_tw

THIS REPORT IS FOR GUIDANCE ONLY. IF USED AS PART OF A REVIEW PROCEDURE FOR PUBLICATION, IT SHOULD NOT REPLACE THE EXPERTISE OF AN EXPERIENCED CRYSTALLOGRAPHIC REFEREE.

No syntax errors found.   CIF dictionary   Interpreting this report

Datablock: zhou_xh_auto_tw

```
Bond precision:   C-C = 0.0134 A          Wavelength=1.54184

Cell:            a=8.9086(4)        b=8.9141(5)       c=18.5488(8)
                 alpha=79.773(4)    beta=87.487(4)    gamma=64.076(5)
Temperature:     150 K

                  Calculated                   Reported
Volume            1302.78(12)                  1302.80(12)
Space group       P 1                          P 1
Hall group        P 1                          P 1
Moiety formula    C15 H19 N O                  C15 H19 N O
Sum formula       C15 H19 N O                  C15 H19 N O
Mr                229.31                       229.31
Dx,g cm-3         1.169                        1.169
Z                 4                            4
Mu (mm-1)         0.564                        0.564
F000              496.0                        496.0
F000'             497.33
h,k,lmax          11,11,23                     10,10,22
Nref              10428[ 5214]                 7137
Tmin,Tmax         0.967,0.972                  0.504,1.000
Tmin'             0.967

Correction method= # Reported T Limits: Tmin=0.504 Tmax=1.000
AbsCorr = MULTI-SCAN
```

Data completeness= 1.37/0.68 Theta(max)= 73.318

R(reflections)= 0.0911(5907) wR2(reflections)= 0.2681(7137)

S = 1.075 Npar= 622

The following ALERTS were generated. Each ALERT has the format
test-name_ALERT_alert-type_alert-level.
Click on the hyperlinks for more details of the test.

Alert level B

```
PLAT340_ALERT_3_B Low Bond Precision on  C-C Bonds ..............    0.01338 Ang.
```

Alert level C

```
ABSTY02_ALERT_1_C  An _exptl_absorpt_correction_type has been given without
            a literature citation. This should be contained in the
            _exptl_absorpt_process_details field.
            Absorption correction given as multi-scan
DIFMX02_ALERT_1_C  The maximum difference density is > 0.1*ZMAX*0.75
            The relevant atom site should be identified.
STRVA01_ALERT_4_C           Flack test results are ambiguous.
           From the CIF: _refine_ls_abs_structure_Flack    0.400
           From the CIF: _refine_ls_abs_structure_Flack_su    0.400
PLAT029_ALERT_3_C _diffrn_measured_fraction_theta_full value Low .      0.965 Why?
PLAT084_ALERT_3_C High wR2 Value (i.e. > 0.25) ..................       0.27 Report
PLAT097_ALERT_2_C Large Reported Max.  (Positive) Residual Density      0.76 eA-3
PLAT250_ALERT_2_C Large U3/U1 Ratio for <U(i,j)> Tensor(Resd    1)       2.2 Note
PLAT911_ALERT_3_C Missing FCF Refl Between Thmin & STh/L=    0.600       162 Report
                8  0  0,   9  0  0,  -9  1  0,  -8  1  0,   8  1  0,   9  1  0,
               10  1  0,  -8  2  0,  -7  2  0,   9  2  0,  10  2  0,  10  3  0,
               -7  4  0,  10  4  0, -10 -4  1,   7 -4  1, -10 -3  1,  -9 -3  1,
              -10 -2  1,  -9 -2  1,   8 -2  1, -10 -1  1,  -9 -1  1,  -8 -1  1,
                8 -1  1,   9 -1  1,  -9  0  1,  -8  0  1,  -7  0  1,   8  0  1,
                   (  132 More Missing: see the .ckf listing file)
```

Alert level G

```
PLAT003_ALERT_2_G Number of Uiso or U(i,j)  Restrained non-H-Atoms        68 Report
PLAT007_ALERT_5_G Number of Unrefined Donor-H Atoms ..............         4 Report
              H001    H007    H003    H002
PLAT032_ALERT_4_G Std. Uncertainty on Flack Parameter Value High .      0.400 Report
PLAT072_ALERT_2_G SHELXL First  Parameter in WGHT  Unusually Large       0.19 Report
PLAT178_ALERT_4_G The CIF-Embedded .res File Contains SIMU Records          3 Report
PLAT188_ALERT_3_G A Non-default SIMU Restraint Value has been used     0.0040 Report
PLAT188_ALERT_3_G A Non-default SIMU Restraint Value has been used     0.0040 Report
PLAT188_ALERT_3_G A Non-default SIMU Restraint Value has been used     0.0040 Report
PLAT720_ALERT_4_G Number of Unusual/Non-Standard Labels ..........        143 Note
              N001    H001    N002    H002    N003    H003    O004    O005
              O006    N007    H007    O008    C009    C00A    C00B    C00C
              H00C    C00D    C00E    H00D    H00E    C00F    C00G    C00H
              H00H    C00I    H00I    C00J    C00K    H00K    C00L    C00M
              H00A    H00B    C00N    H00N    C00O    H00O    C00P    H00P
```

```
               C00Q    C00R    C00S    H00S    C00T    H00L    H00M    C00U
               H00U    C00V    H00V    C00W    H00F    H00G    H00J    C00X
               H00X    C00Y    C00Z    H00Z    C010    C011    H011    C012
               H012    C013    H013    C014    H014    C015    H015    C016
               H016    C017    H017    C018    C019    C01A    C01B    H01L
               C01C    H01A    H01B    H01C    C01D    H01W    H01X    C01E
               H1AA    C01F    H01F    C01G    H01Y    C01H    H01Z    C01I
               H2AA    Ha      C01J    H3AA    Hb      C01K    H01D    H01E
               C01L    C01M    H01M    H01N    C01N    H01     Hc      C01O
               H4AA    C01P    H01G    H01H    C01Q    H01I    H01J    H01K
               C01R    H01O    H01P    C01S    H0AA    Hd      He      C01T
               H01Q    H01R    H01S    C01U    H01T    H01U    H01V    C01V
               H5AA    Hf      Hg      C01W    H6AA    Hh      Hi
PLAT791_ALERT_4_G Model has Chirality at C00N       (Sohncke SpGr)          R Verify
PLAT791_ALERT_4_G Model has Chirality at C00S       (Sohncke SpGr)          S Verify
PLAT791_ALERT_4_G Model has Chirality at C012       (Sohncke SpGr)          R Verify
PLAT791_ALERT_4_G Model has Chirality at C015       (Sohncke SpGr)          S Verify
PLAT860_ALERT_3_G Number of Least-Squares Restraints .............       1011 Note
PLAT870_ALERT_4_G ALERTS Related to Twinning Effects Suppressed ..          ! Info
PLAT910_ALERT_3_G Missing FCF Reflection(s) Below Theta(Min)[Deg]=       4.85 Note
                0  0  1,
PLAT912_ALERT_4_G Missing # of FCF Reflections Above STh/L=  0.600        192 Note
PLAT931_ALERT_5_G CIFcalcFCF  Twin Law ( 2-2-1)         Est.d BASF       0.53 Check
PLAT941_ALERT_3_G Average HKL Measurement Multiplicity ...........        1.5 Low
PLAT969_ALERT_5_G The 'Henn et al.' R-Factor-gap value ...........      4.250 Note
               Predicted wR2: Based on SigI**2  6.31 or SHELX Weight 24.94
```

```
  0 ALERT level A = Most likely a serious problem - resolve or explain
  1 ALERT level B = A potentially serious problem, consider carefully
  8 ALERT level C = Check. Ensure it is not caused by an omission or oversight
 20 ALERT level G = General information/check it is not something unexpected

  2 ALERT type 1 CIF construction/syntax error, inconsistent or missing data
  4 ALERT type 2 Indicator that the structure model may be wrong or deficient
 10 ALERT type 3 Indicator that the structure quality may be low
 10 ALERT type 4 Improvement, methodology, query or suggestion
  3 ALERT type 5 Informative message, check
```

It is advisable to attempt to resolve as many as possible of the alerts in all categories. Often the minor alerts point to easily fixed oversights, errors and omissions in your CIF or refinement strategy, so attention to these fine details can be worthwhile. It is up to the individual to critically assess their own results and, if necessary, seek expert advice.

PLATON version of 26/09/2025; check.def file version of 20/09/2025

---

duplicate check

No duplication found

Datablock zhou_xh_auto_tw - ellipsoid plot

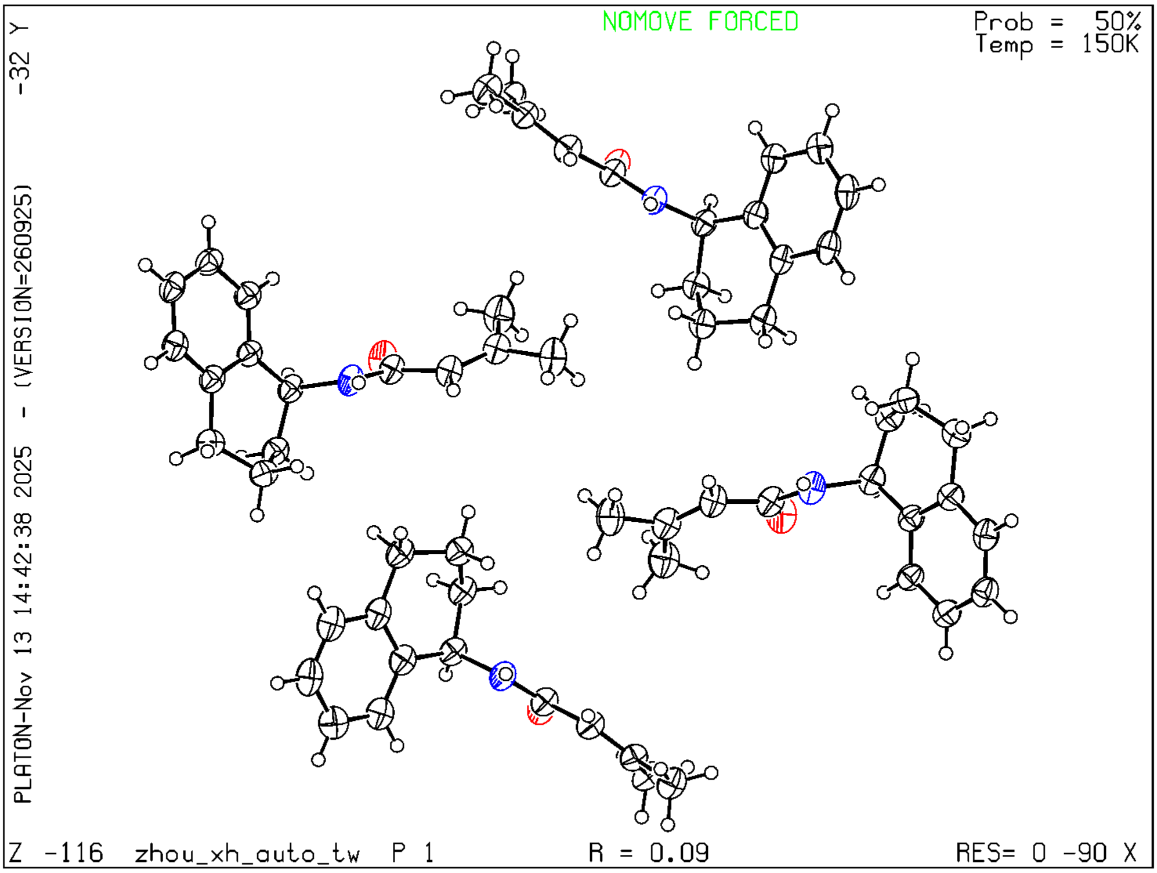

Supplement: Supplementary file 1 [file biology-15-02003-s001.zip › Replace the originally submitted Supplementary Materials/File S2/3-Methyl-N-(1,2,3,4-tetrahydronaphthalen-1-yl)but-2-enamide.pdf]
