# Supplementary material for: Discovery of Bioactive N-Substituted-3-methylbut-2-enamides: Synthesis, Crystal Structures, Antifungal Activity, Mechanism of Action, and Zebrafish Toxicity Evaluation
Source: Biology (Basel). 2026 Nov 4;15(21):2003. doi: 10.3390/biology15212003 (PMC13761460; doi:10.3390/biology15212003)

checkCIF/PLATON report

You have not supplied any structure factors. As a result the full set of tests cannot be run.

THIS REPORT IS FOR GUIDANCE ONLY. IF USED AS PART OF A REVIEW PROCEDURE FOR PUBLICATION, IT SHOULD NOT REPLACE THE EXPERTISE OF AN EXPERIENCED CRYSTALLOGRAPHIC REFEREE.

No syntax errors found. CIF dictionary Interpreting this report

Datablock: zhou_m_auto

```
Bond precision:   C-C = 0.0023 A          Wavelength=1.54184

Cell:              a=5.47422(7)      b=9.58669(12)      c=29.2280(4)
                   alpha=90          beta=90            gamma=90
Temperature:       150 K

                 Calculated                   Reported
Volume           1533.88(3)                   1533.87(3)
Space group      P 21 21 21                   P 21 21 21
Hall group       P 2ac 2ab                    P 2ac 2ab
Moiety formula   C18 H19 N O2                 C18 H19 N O2
Sum formula      C18 H19 N O2                 C18 H19 N O2
Mr               281.34                       281.34
Dx,g cm-3        1.218                        1.218
Z                4                            4
Mu (mm-1)        0.629                        0.629
F000             600.0                        600.0
F000'            601.73
h,k,lmax         6,11,36                      6,11,35
Nref             3065[ 1814]                  2956
Tmin,Tmax        0.904,0.927                  0.793,1.000
Tmin'            0.904

Correction method= # Reported T Limits: Tmin=0.793 Tmax=1.000
AbsCorr = MULTI-SCAN
```

```
Data completeness= 1.63/0.96        Theta(max)= 72.926

R(reflections)= 0.0287( 2902)       wR2(reflections)= 0.0744( 2956)

S = 1.082                  Npar= 196
```

```
The following ALERTS were generated. Each ALERT has the format
       test-name_ALERT_alert-type_alert-level.
Click on the hyperlinks for more details of the test.
```

Alert level G

```
PLAT994_ALERT_1_G SHELXL .ins Contains no or MERG 0 Instruction ..          ! Note
```

```
   0 ALERT level A = Most likely a serious problem - resolve or explain
   0 ALERT level B = A potentially serious problem, consider carefully
   0 ALERT level C = Check. Ensure it is not caused by an omission or oversight
   1 ALERT level G = General information/check it is not something unexpected

   1 ALERT type 1 CIF construction/syntax error, inconsistent or missing data
   0 ALERT type 2 Indicator that the structure model may be wrong or deficient
   0 ALERT type 3 Indicator that the structure quality may be low
   0 ALERT type 4 Improvement, methodology, query or suggestion
   0 ALERT type 5 Informative message, check
```

It is advisable to attempt to resolve as many as possible of the alerts in all categories. Often the minor alerts point to easily fixed oversights, errors and omissions in your CIF or refinement strategy, so attention to these fine details can be worthwhile. It is up to the individual to critically assess their own results and, if necessary, seek expert advice.

PLATON version of 15/01/2026; check.def file version of 02/01/2026

---

duplicate check

No duplication found

Datablock zhou_m_auto - ellipsoid plot

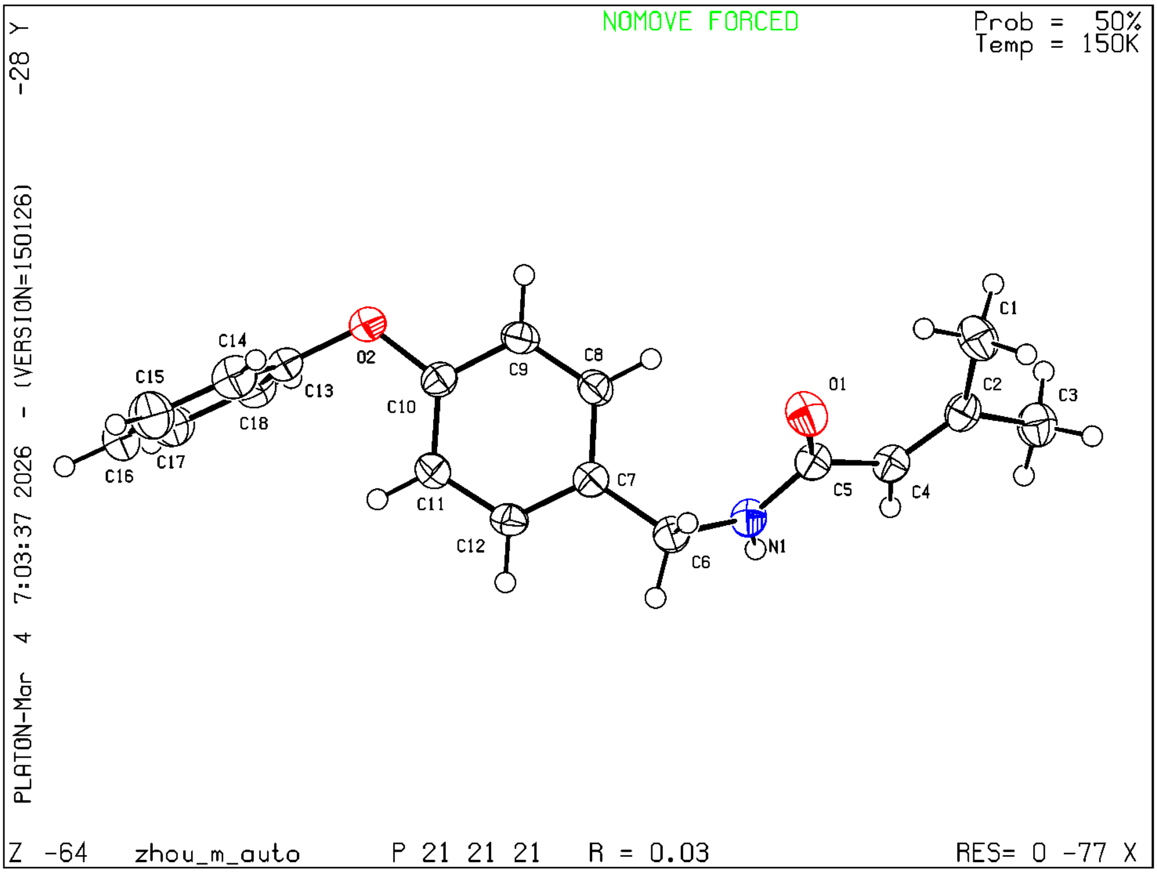

Supplement: Supplementary file 1 [file biology-15-02003-s001.zip › Replace the originally submitted Supplementary Materials/File S2/3‐Methyl‐N‐(4‐phenoxybenzyl)but‐2‐enamide.pdf]
