# Supplementary material for: Discovery of Bioactive N-Substituted-3-methylbut-2-enamides: Synthesis, Crystal Structures, Antifungal Activity, Mechanism of Action, and Zebrafish Toxicity Evaluation
Source: Biology (Basel). 2026 Nov 4;15(21):2003. doi: 10.3390/biology15212003 (PMC13761460; doi:10.3390/biology15212003)

checkCIF/PLATON report

Structure factors have been supplied for datablock(s) zhou_tx20_auto

THIS REPORT IS FOR GUIDANCE ONLY. IF USED AS PART OF A REVIEW PROCEDURE FOR PUBLICATION, IT SHOULD NOT REPLACE THE EXPERTISE OF AN EXPERIENCED CRYSTALLOGRAPHIC REFEREE.

No syntax errors found. CIF dictionary Interpreting this report

Datablock: zhou_tx20_auto

```
Bond precision:   C-C = 0.0019 A          Wavelength=1.54184

Cell:             a=11.6805(3)     b=11.3228(2)        c=9.7294(2)
                  alpha=90         beta=114.000(3)     gamma=90
Temperature:      120 K

                 Calculated                   Reported
Volume           1175.52(5)                   1175.53(5)
Space group      P 21/c                       P 1 21/c 1
Hall group       -P 2ybc                      -P 2ybc
Moiety formula   C14 H17 N O                  C14 H17 N O
Sum formula      C14 H17 N O                  C14 H17 N O
Mr               215.29                       215.28
Dx,g cm-3        1.217                        1.216
Z                4                            4
Mu (mm-1)        0.594                        0.594
F000             464.0                        464.0
F000'            465.26
h,k,lmax         14,14,12                     14,14,11
Nref             2349                         2201
Tmin,Tmax        0.918,0.942                  0.864,1.000
Tmin'            0.915

Correction method= # Reported T Limits: Tmin=0.864 Tmax=1.000
AbsCorr = MULTI-SCAN
```

Data completeness= 0.937 Theta(max)= 72.938

R(reflections)= 0.0380(2094) wR2(reflections)= 0.1028(2201)

S = 1.050 Npar= 152

The following ALERTS were generated. Each ALERT has the format
test-name_ALERT_alert-type_alert-level.
Click on the hyperlinks for more details of the test.

Alert level C

```
PLAT029_ALERT_3_C _diffrn_measured_fraction_theta_full value Low .      0.975 Why?
PLAT911_ALERT_3_C Missing FCF Refl Between Thmin & STh/L=    0.600         53 Report
               10  0  4,  10  1  4,   9  1  5,   7  0  6,   8  0  6,   8  1  6,
                6  1  7,   2  0  8,   3  0  8,   4  0  8,   5  0  8,   2  1  8,
                3  1  8,   4  1  8,   5  1  8,   4  2  8,   5  2  8,   0  1  9,
                1  1  9,   2  1  9,   3  1  9,   2  2  9,   3  2  9,   2  3  9,
                3  3  9,  -3  0 10,  -2  0 10,  -1  0 10,   0  0 10,   1  0 10,
                   (   23 More Missing: see the .ckf listing file)
```

Alert level G

```
PLAT793_ALERT_4_G Model has Chirality at C7          (Centro SpGr)          R Verify
PLAT912_ALERT_4_G Missing # of FCF Reflections Above STh/L=  0.600         90 Note
PLAT969_ALERT_5_G The 'Henn et al.' R-Factor-gap value ..........      11.101 Note
              Predicted wR2: Based on SigI**2  0.93 or SHELX Weight  9.79
PLAT978_ALERT_2_G Number C-C Bonds with Positive Residual Density.         10 Info
```

---

0 **ALERT level A** = Most likely a serious problem - resolve or explain
0 **ALERT level B** = A potentially serious problem, consider carefully
2 **ALERT level C** = Check. Ensure it is not caused by an omission or oversight
4 **ALERT level G** = General information/check it is not something unexpected

0 ALERT type 1 CIF construction/syntax error, inconsistent or missing data
1 ALERT type 2 Indicator that the structure model may be wrong or deficient
2 ALERT type 3 Indicator that the structure quality may be low
2 ALERT type 4 Improvement, methodology, query or suggestion
1 ALERT type 5 Informative message, check

It is advisable to attempt to resolve as many as possible of the alerts in all categories. Often the minor alerts point to easily fixed oversights, errors and omissions in your CIF or refinement strategy, so attention to these fine details can be worthwhile. It is up to the individual to critically assess their own results and, if necessary, seek expert advice.

PLATON version of 26/09/2025; check.def file version of 20/09/2025

---

duplicate check

No duplication found

Datablock zhou_tx20_auto - ellipsoid plot

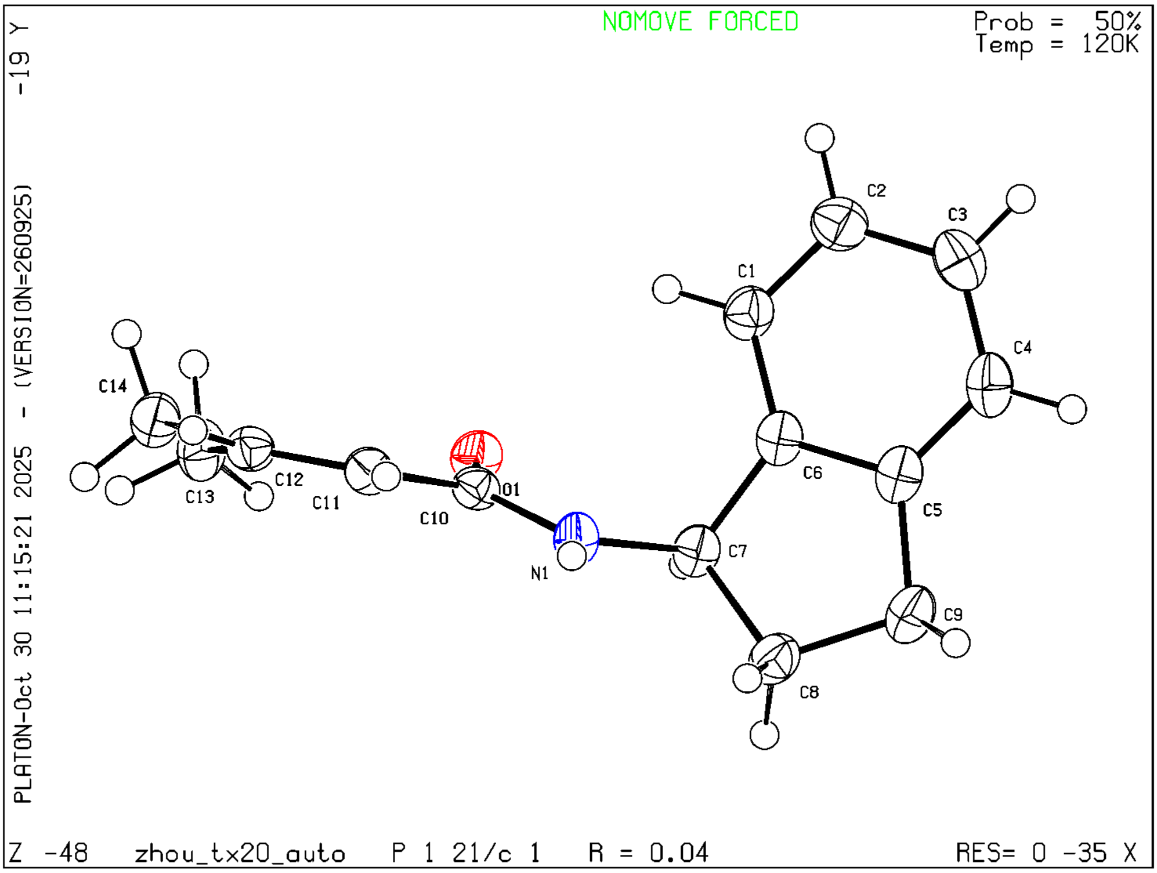

Supplement: Supplementary file 1 [file biology-15-02003-s001.zip › Replace the originally submitted Supplementary Materials/File S2/N-(2,3-Dihydro-1H-inden-1-yl)-3-methylbut-2-enamide.pdf]
